# Comparable efficacy and mechanisms of sterile soil ingestion versus low hygiene exposure in DSS-induced colitis

**DOI:** 10.1128/aem.02415-25

**Published:** 2026-02-09

**Authors:** Mengjie Li, Yifan Feng, Xiaoyuan Cao, Na Li, Xuanchen Chen, Zhimao Bai, Zhongjie Fei, Zuhong Lu, Honglin Zhang, Dongrui Zhou

**Affiliations:** 1 Key Laboratory of Child Development and Learning Science of Ministry of Education, Southeast University 12579, Nanjing, China; 2 School of Life Science and Technology, Southeast University 12579, Nanjing, China; 3 College of Food Science, Nanjing Xiaozhuang University, Nanjing, China; 4 Key Laboratory of Environmental Medicine Engineering of Ministry of Education, Southeast University 12579, Nanjing, China; 5 Key Laboratory of Animal Diseases Diagnostic and Immunology, College of Veterinary Medicine, Nanjing Agricultural University 261674, Nanjing, China; 6 State Key Laboratory of Bioelectronics, School of Biological Science and Medical Engineering, Southeast University 12579, Nanjing, Jiangsu, China; Norwegian University of Life Sciences, Ås, Norway

**Keywords:** ulcerative colitis, gut microbiota, low hygiene exposure, soil, immune

## Abstract

Accumulating evidence suggests that exposure to low-hygiene environments (LHE) can alleviate chronic diseases by modulating the gut microbiota. Soil has been identified as a key factor in shaping microbial communities within the LHE. Furthermore, influenced by cultural customs and traditions, the practice of soil ingestion persists in many regions worldwide, serving both nutritional and medicinal purposes. However, whether soil confers efficacy comparable to that of LHE exposure or which intervention demonstrates better efficacy remains unexplored. This study compared the interventional effects and underlying mechanisms of sterile soil ingestion versus LHE exposure in dextran sulfate sodium (DSS)-induced chronic ulcerative colitis (UC) mice. Our results demonstrated that UC mice exhibited significant gut dysbiosis, characterized by reduced microbial diversity and disruption of microbial network structure. Both LHE exposure and soil ingestion markedly reshaped the gut microbial ecosystem, exhibiting highly similar regulatory patterns in microbial composition. LHE exposure increased fecal acetic acid concentrations, while soil ingestion elevated butyric acid levels. Moreover, both interventions effectively alleviated UC clinical symptoms, restored intestinal barrier integrity, and downregulated pro-inflammatory cytokine levels. In summary, sterile soil ingestion and LHE exposure exert comparable protective effects in UC mice. Given its operational simplicity and feasibility, sterile soil represents a promising translation and application. These findings support the potential of sterile soil as a practical therapeutic approach for alleviating UC-related gut ecological disruption in modern urban settings.

**IMPORTANCE:**

Urbanization and excessive hygiene have reduced human exposure to soil and environmental microorganisms, contributing to the rising incidence of immune-related disorders such as inflammatory bowel disease. This study demonstrates that sterile soil ingestion and low-hygiene environment exposure comparably reshape the gut microbiota, enhance short-chain fatty acid production, and alleviate colitis in mice. These findings highlight sterile soil as a practical and controllable intervention to mimic the protective benefits observed in traditional, microbe-rich environments. Given the challenges of accessing low-hygiene settings in modern urban life, sterile soil represents a feasible therapeutic approach to alleviate gut dysbiosis and inflammation, with broad implications for microbiome-based therapeutics in industrialized societies.

## INTRODUCTION

Ulcerative colitis (UC), a major subtype of inflammatory bowel disease (IBD), involves a complex interplay of environmental, microbial, immune, and genetic factors (1, 2). Increasing UC incidence parallels rapid transformations in modern lifestyles, dietary patterns, and environmental conditions (3). Epidemiological data reveal higher UC prevalence in Western countries, with emerging industrial countries rapidly increasing rates following accelerated economic development and Westernization (4, 5). Notably, descendants of immigrants from low-IBD-incidence Southeast Asian countries to high-incidence Western nations exhibit disease risk comparable to non-immigrant populations (6, 7). While the reasons for regional differences in UC prevalence remain unclear, evidence implicates that Westernization-associated environmental factors, particularly over sanitation, may alter mucosal immune responses to the gut microbiota in genetically susceptible individuals (8). These findings underscore the critical role of environmental factors in UC pathogenesis.

Gut microbial dysbiosis plays a key role in the pathogenesis of UC. Research indicates that alterations in environmental or host factors can induce disruption of the intestinal microbiota, thereby compromising homeostatic interactions between gut microbiota and the host, and ultimately driving the progression of IBD (9, 10). UC patients exhibit significant reductions in microbial diversity and stability, and this loss of diversity may compromise intestinal barrier function and impair immune regulatory capacity, thereby triggering inflammatory and immune responses (11).

Research demonstrates that urbanization-driven lifestyle changes—including high-fat diets, antibiotic overuse, and reduced environmental microbes exposure—significantly decrease gut microbiota diversity while increasing susceptibility to diseases such as IBD (12). Multiple studies support that rural-urban environmental and lifestyle differences may explain the higher IBD incidence in urban populations (12–14). Accumulating evidence indicates that low-hygiene environments (LHE) can reshape gut microbiota, enhance immune regulatory functions, effectively promote resistance to asthma and eczema (15, 16), and accelerate recovery from antibiotic-induced gut dysbiosis (17). Although these findings highlight the potential health benefits of LHE, accelerating urbanization has progressively distanced populations from traditional farming lifestyles, making such exposure increasingly difficult in modern cities. Therefore, identifying key protective components within LHE is crucial, as this will establish theoretical and practical foundations for promoting health through artificial replication or supplementation of these elements.

Sterile soil, rather than its microbial components, plays a key role in maintaining gut microbial diversity and health. Studies have demonstrated that sterile soil ingestion significantly increased gut microbial diversity while effectively alleviating allergic responses (18). Furthermore, sterile soil intervention reversed antibiotic-induced gut dysbiosis, ameliorating behavioral abnormalities and restoring intestinal barrier function (19). Additional studies reveal that sterile soil conferred protection against asthma by modulating gut microbiota and immune homeostasis (20). Collectively, these findings indicate that even in the absence of viable microorganisms, the soil’s physicochemical components actively reshape gut microecosystems and regulate host immunity, thereby exerting protective effects against immune-related disorders. This insight establishes a novel theoretical foundation for maintaining gut ecological health in the context of modern urbanization and provides critical groundwork for developing soil-derived functional products.

Influenced by traditional culture and customs, geophagy (soil consumption) is a phenomenon with a long history and wide global distribution. This practice remains relatively common in many regions, including Asia, Africa, South America, and Latin America (21). For example, pregnant women often consume soil to supplement minerals or alleviate pregnancy-related discomforts (22). In traditional medicine, specific types of soil, such as kaolin and montmorillonite, are used to treat gastrointestinal disorders and exhibit properties such as antibacterial and antiviral effects (23). Additionally, geophagy may modulate immune responses and introduce beneficial microorganisms into the gut microbiota (24). In certain parts of China, such as Henan and Shanxi, there is a custom of incorporating kaolin into flour-based foods, with the intention of supplementing trace elements while promoting digestion and intestinal health. A recent study reported that a montmorillonite-based biomimetic material alleviated gut microbiota disorders and reduced inflammation in mice with colitis (25).

To evaluate the therapeutic effects of the interventions on UC, we analyzed key genes involved in intestinal barrier integrity and immune regulation. Tight junction proteins (ZO-1, Claudins) were assessed to determine changes in epithelial barrier function, whose disruption is a hallmark of UC (26, 27). Pro-inflammatory cytokines (IL-1β, IL-6, TNF-α, and IL-17A) were measured as their elevated levels are closely associated with disease severity in UC (28, 29), while IL-10 was evaluated as a pivotal anti-inflammatory cytokine that maintains intestinal immune tolerance and suppresses excessive inflammation (30). Additionally, IFN-γ and IL-4 were analyzed as representative cytokines of Th1 and Th2 immune responses, respectively, whose imbalance plays a critical role in the pathogenesis of IBD (31).

Although existing studies have confirmed that both LHE and sterile soil ingestion can modulate gut microbiota and immune function, systematic comparisons between these two strategies remain lacking. To address this gap, we established three murine housing environments with graded hygienic status: specific pathogen-free (SPF) conditions, low-hygiene environment, and conventional animal facility environment (with hygiene levels intermediate between the former two, simulating modern human living conditions). Additionally, to evaluate the potential role of soil intervention in UC therapy, we supplemented 5% sterile soil into the diet of mice housed under conventional animal facility conditions.

## MATERIALS AND METHODS

### Animals and the chronic UC model

Adult SPF C57BL/6 mice were purchased from B & K Universal (Shanghai, China) and housed at the Southeast University Laboratory Animal Center SPF facility. Breeding pairs were established at a 1:3 (male: female) ratio for mating. Offspring at 3–4 weeks of age were randomly allocated into four experimental groups: Control group (Control, *n* = 10), dextran sulfate sodium (DSS)-induced UC model group (DSS, *n* = 9), low-hygiene environment exposure group (XZ, *n* = 9), and sterile soil intervention group (Soil, *n* = 9). When mice reached 6 weeks of age, DSS-induced chronic colitis modeling was initiated.

**Fig 1:**
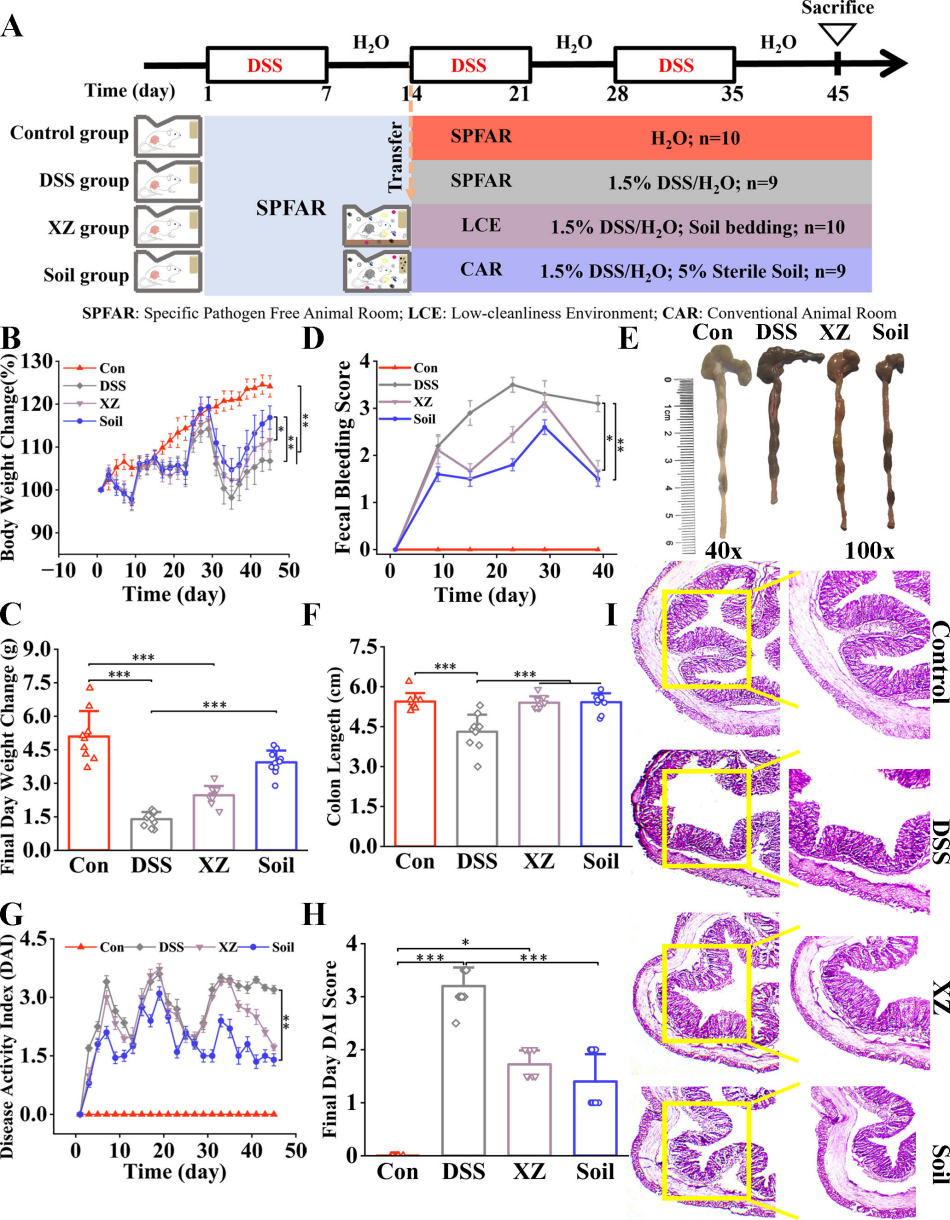
LHE exposure and sterile soil ingestion alleviate symptoms in UC mice. (**A**) Schematic overview of experiment design. (**B**) Percentage change in body weight during the experimental period. (**C**) Body weight change on the final experimental day. (**D**) Fecal bleeding scores. (**E**) Representative photographs of colons. (**F**) Colon length change. (**G**) DAI scores. (**H**) DAI scores on the final experimental day. (**I**) Representative H&E staining of colon. *n* = 9–10. Data are presented as mean ± SEM. Statistical significance was determined by one-way ANOVA or Kruskal-Wallis test with Bonferroni’s *post hoc* test for multiple comparisons. *P < 0.05, **P < 0.01, ***P < 0.001.

The chronic UC mouse modeling protocol was as follows: 7 days of 1.5% DSS (MP Biomedicals, USA) administration followed by 7 days of sterile water; this process was repeated for three cycles. Control mice received sterile water throughout the study. The low-hygiene environment for the XZ group was established following the protocol described by Zhou et al. (15), with bedding material comprising 25 g house dust, 25 g decaying hay, 25 g decaying fallen leaves, and 25 g soil (0–10 cm depth). All materials were collected from a well-vegetated, undisturbed area on the campus of Nanjing Xiaozhuang University, which has no history of agricultural or industrial activity. Following the first DSS/water cycle, both XZ and Soil groups were transferred to the same conventional animal room. Within this facility, XZ mice were housed in low-hygiene conditions, while the Soil group received standard feed supplemented with 5% sterile soil and maintained on sterile bedding. Control and DSS groups remained in SPF conditions. After the final intervention cycle, fecal samples were collected. Mice were euthanized the following day, with colon tissues collected for subsequent experiments. A schematic of the experimental timeline and intervention design is illustrated in Fig. 1A.

### Animal housing environments

Mice were housed in either an SPF barrier facility with an independent filtered air supply and exhaust, ensuring a defined pathogen-free status and minimal environmental microbial exposure beyond the animals’ own gut and cage microbiota, or a conventional open animal room permitting air exchange with the external environment with environmental microorganisms. All other conditions (temperature: 24 ± 2 ℃; humidity: 40%–60%; 12 h light/dark cycle) were identical. Photographs of both facilities are provided in Fig. S1.

### Fecal sample collection and storage

Fecal samples were collected within a super-clean bench in the animal facility. The collection procedure was as follows: Mice were gently lifted by the tail to expose the anus, facilitating spontaneous defecation. As feces were about to be expelled, a sterile 1.5 mL microcentrifuge tube was swiftly positioned to allow the pellets to fall directly into the tube. Approximately 4–5 fecal pellets were collected per mouse. In cases where a mouse did not defecate promptly, the collection attempt was paused, and the sample tube was temporarily stored on dry ice. A new tube was used for a subsequent collection attempt from the same mouse. All samples were transferred to a −80°C freezer on the same day for long-term storage.

### Soil and feed

The standard laboratory feed was purchased from Jiangsu Xietong Pharmaceutical Bio-engineering Co., Ltd (Nanjing, China). Surface soil samples (0–10 cm depth) were collected from a well-vegetated and ecologically preserved area on the campus of Nanjing Xiaozhuang University, which has no history of agricultural or industrial activity. The chemical composition of the soil was determined using a Wavelength Dispersive X-Ray Fluorescence spectrometer (18) (Thermo Fisher Scientific, USA). The data are provided in Table S1. Following natural air-drying, the soil was thoroughly homogenized by grinding and sieved through a 100-mesh sieve. The processed soil was then subjected to triple autoclave sterilization (121°C for 30 min per cycle) to effectively eliminate viable microorganisms. Sterilized soil was mixed with pre-ground standard feed at a 5:95 (wt/wt) ratio. During mixing, sterile water was added to reconstitute the mixture into pellets. All procedures were conducted in a clean bench to ensure sterility of the soil-supplemented diet.

### Disease activity index score

The DAI score was used to evaluate the UC severity and was calculated as the average score of three parameters: weight loss, stool consistency, and fecal occult blood. The scoring criteria were as follows: weight loss/% (<1, 0; 1–5, 1; 6–10, 2; 100–15, 3; >15, 4), fecal consistency (normal, 0; loose stools, 1; semi loose stool, 2; stools of irregular shape, 3; diarrhea, 4), fecal occult blood (no rectal bleeding, 0; presence of blood, 1; slight hematochezia, 2; mild bleeding, 3; serious bleeding, 4).

### Reverse transcription-quantitative PCR

All materials were treated with diethyl pyrocarbonate (DEPC, Biomiga, USA) to eliminate RNase contamination. Total RNA was extracted using an RNA extraction kit (Yugong Biolabs, China) according to the manufacturer’s protocols. The purity of the total RNA was measured on a Nanophotometer (Implen, Germany). cDNA synthesis was performed using a reverse transcription kit (Yugong Biolabs, China). cDNA amplification was performed using the SLAN 96S PCR System (Hongshi, China) with gene-specific primers (Table S2), following the protocol of the Taq SYBR Green qPCR Premix (Universal) (Biomiga, USA). Amplification conditions were as follows: 30 s at 95°C, 40 cycles of 10 s at 95°C, 10 s at 60°C, and 30 s at 72°C. Relative gene expression levels were calculated using the 2^−ΔΔCT^. Glyceraldehyde 3-phosphate dehydrogenase (GAPDH) was used as a reference gene.

### 16S rRNA gene sequencing

Mouse fecal bacterial DNA was extracted using the E.Z.N.A. DNA Kit (Omega Bio-tek, USA) following the manufacturer’s instructions. The V4-V5 region of the bacteria 16S ribosomal RNA gene was amplified by PCR using primers 515F 5′-barcode-GTGCCAGCMGCCGCGG-3′ and 907R 5′-CCGTCAATTCMTTTRAGTTT-3′. Subsequently, the purified PCR products were quantified using the Qubit3.0 (Life Invitrogen). The pooled DNA was then used to construct Illumina Pair-End library following Illumina’s genomic DNA library preparation protocol. The amplicon library was subjected to paired-end sequencing (2 × 250) on an Illumina MiSeq platform (Shanghai BIOZERON Co., Ltd) following standard procedures.

### Detection of SCFAs content

The sample preparation and detection methods for short-chain fatty acids (SCFAs) in mouse feces were adapted from Zhao et al. (32). Fifty milligrams of mouse feces was dissolved in 100 mL of distilled water. After thorough dissolution, the mixture was centrifuged at 6,000 × *g* for 5 min. Then, 500 μL of the supernatant was collected and mixed with 10 μL of 2-ethylbutyric acid (diluted 4 × 10^5^ fold) as an internal standard. After extraction, the analytes were eluted with 100 μL of elution solution, which was prepared by adding 15 μL of concentrated hydrochloric acid to 15 mL of ethanol. Solid-phase microextraction was subsequently performed using activated polypyrrole nanofibers. Quantitative analysis of SCFAs was performed using a gas chromatography-mass spectrometry (GC-MS) system (Thermo, USA). Chromatographic separation was achieved using a fused-silica capillary column (30 m × 0.32 mm i.d.) coated with a 0.5 μm polyethylene glycol phase (DB-WAX, J&W Scientific, Agilent Technologies Inc., USA). Helium was used as the carrier gas at a flow rate of 1.5 mL/min. The temperature program was as follows: initial temperature of 60°C held for 1 min; ramped at 10°C/min to 110°C and held for 5 min; then ramped at 3°C/min to 161 °C and held for 5 min.

### Statistical analysis

Microbiome data were processed and analyzed using QIIME 2 software, primarily for quality control of raw sequencing data, feature extraction, and diversity analysis. To construct the microbial community correlation networks, low-abundance OTUs were filtered before analysis. Associations with *P*-values < 0.05 and correlation coefficients > 0.6 were selected for network generation. Networks were visualized in Gephi (v. 0.10.1) using the Fruchterman-Reingold layout algorithm. Correlation analysis between immune factors and microbial communities was conducted using the Mantel test and visualized through the ChiPlot online platform (https://www.chiplot.online). Heatmaps were generated using the *pheatmap* package in R.

Group comparisons employed one-way ANOVA (normally distributed data) or Kruskal-Wallis test (non-parametric data) with Bonferroni correction for multiple comparisons. Data are presented as mean ± SEM. Graphical visualizations were created in Origin 2025. *P* < 0.05 was considered statistically significant.

## RESULTS

### Sterile soil intake and LHE exposure alleviate UC symptoms

DSS-treated mice exhibited significant physiological alterations compared to healthy controls. During the experimental period, DSS administration induced pronounced weight loss, diarrhea, and fecal bleeding compared to the Control mice (Fig. 1B through D). In addition, the model group showed significantly shortened colon length and elevated DAI scores (Fig. 1E through H). Both LHE exposure and sterile soil supplementation effectively alleviated these clinical symptoms.

Histopathological analysis revealed distinct morphological characteristics among experimental groups. Control mice exhibited intact colonic architecture with well-preserved crypt structures and absence of inflammatory infiltration (Fig. 1I). In contrast, DSS-treated mice displayed typical pathological features of colitis, including partial loss of crypts and goblet cells, epithelial denudation, and marked inflammatory cell infiltration. Following intervention with either LHE exposure or sterile soil ingestion, significant amelioration of these pathological alterations was observed, with the tissue structure appearing closer to normal and inflammatory damage significantly reduced. These results indicate that both intervention strategies effectively improved DSS-induced UC symptoms.

### Gut dysbiosis induced by UC

**Fig 2:**
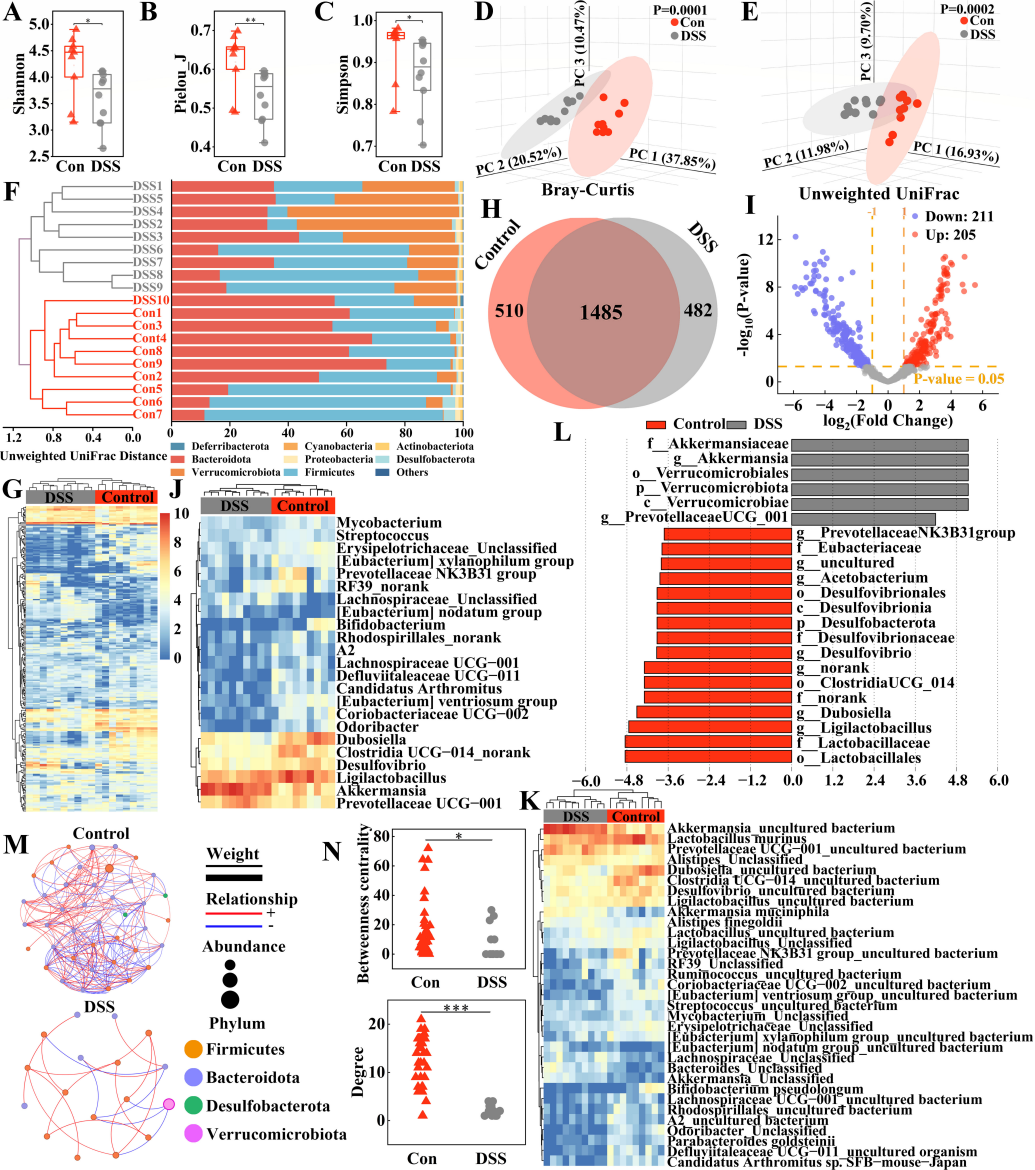
DSS-induced gut microbial dysbiosis. (**A**) Shannon index. (**B**) Pielou_J index. (**C**) Simpson index. (**D**) PCoA based on Bray-Curtis distance. (**E**) PCoA based on Unweighted UniFrac distance. (**F**) Unweighted UniFrac distance-based clustering tree (left) and relative abundance of bacterial phyla (right). (**G**) Hierarchical clustering heatmap of the top 250 OTUs. (**H**) OTU Venn diagram. (**I**) Volcano plot showing differential OTUs. (**J**) Heatmap of differential genera. (**K**) Heatmap of differential species. (**L**) LEfSe identifying differentially abundant taxa. LDA score > 3.6. (**M**) Microbial correlation network. Node colors represent bacterial phyla. Node sizes indicate OTU abundance. Red edges indicate positive correlations; blue edges indicate negative correlations. Edge thickness reflects the magnitude of correlation coefficients. (**N**) Comparison of degree and betweenness centrality. Statistical differences were analyzed using independent-sample *t*-tests. *P < 0.05, **P < 0.01, ***P < 0.001.

Gut dysbiosis is considered one of the key factors influencing UC development. To investigate the impact of DSS-induced UC on the gut microbiota, we first systematically analyzed the gut microbial characteristics of model and control mice. The results showed that DSS treatment significantly reduced Shannon index, Pielou_J’s index, and Simpson index (Fig. 2A through C; Table S3), indicating decreased microbial community diversity and evenness. Beta diversity analysis further revealed significant intergroup differences. Principal Coordinates Analysis (PCoA) based on both unweighted UniFrac distance and Bray-Curtis distance demonstrated clear clustering separation between the Control and DSS groups (Fig. 2D and E). Similarly, phylogenetic tree analysis also displayed distinct branching structures (Fig. 2F).

At the phylum level, UC mice exhibited a significant increase in the abundance of Verrucomicrobiota and a significant decrease in Desulfobacterota (Fig. 2F; Fig. S2A and B; Table S4). Besides, significant alterations were observed in the genus composition of the phyla Proteobacteria, Bacteroidota, and Firmicutes (Fig. S2C through E). Clustering heatmap analysis based on the top 250 OTUs revealed distinct OTU abundance profiles between the DSS and Control groups (Fig. 2G). Venn diagram analysis identified 1,485 OTUs shared between groups, with 510 and 482 OTUs unique to the Control and DSS groups, respectively (Fig. 2H). Furthermore, 416 differentially abundant OTUs were identified between groups, with 211 downregulated and 205 upregulated in the DSS group (Fig. 2I). Differential abundance heatmaps at the genus and species levels demonstrated that DSS treatment significantly reduced the abundance of most key bacterial genera and species (Fig. 2J and K; Tables S5 and S6). Linear Discriminant Analysis Effect Size (LEfSe) analysis identified *Ligilactobacillus* and *Dubosiella* as characteristic genera of the Control group, while *Akkermansia* was significantly enriched in the DSS group (Fig. 2L; Fig. S2F). Furthermore, UC mice showed remarkable simplification of microbial interaction networks, characterized by a 60.9% reduction in nodes (17 vs 41), 92.6% decrease in connections (19 vs 256), and significant declines in topological parameters, including degree and betweenness centrality, indicating a loss of microbial community connectivity and stability (Fig. 2M and N). These results indicate that UC not only caused loss of gut microbial diversity and structural dysbiosis but also severely disrupted microbial interaction networks, leading to significantly reduced stability and complexity of the gut microbiota.

### Comparable impacts of soil ingestion and LHE exposure on gut microbiota

**Fig 3:**
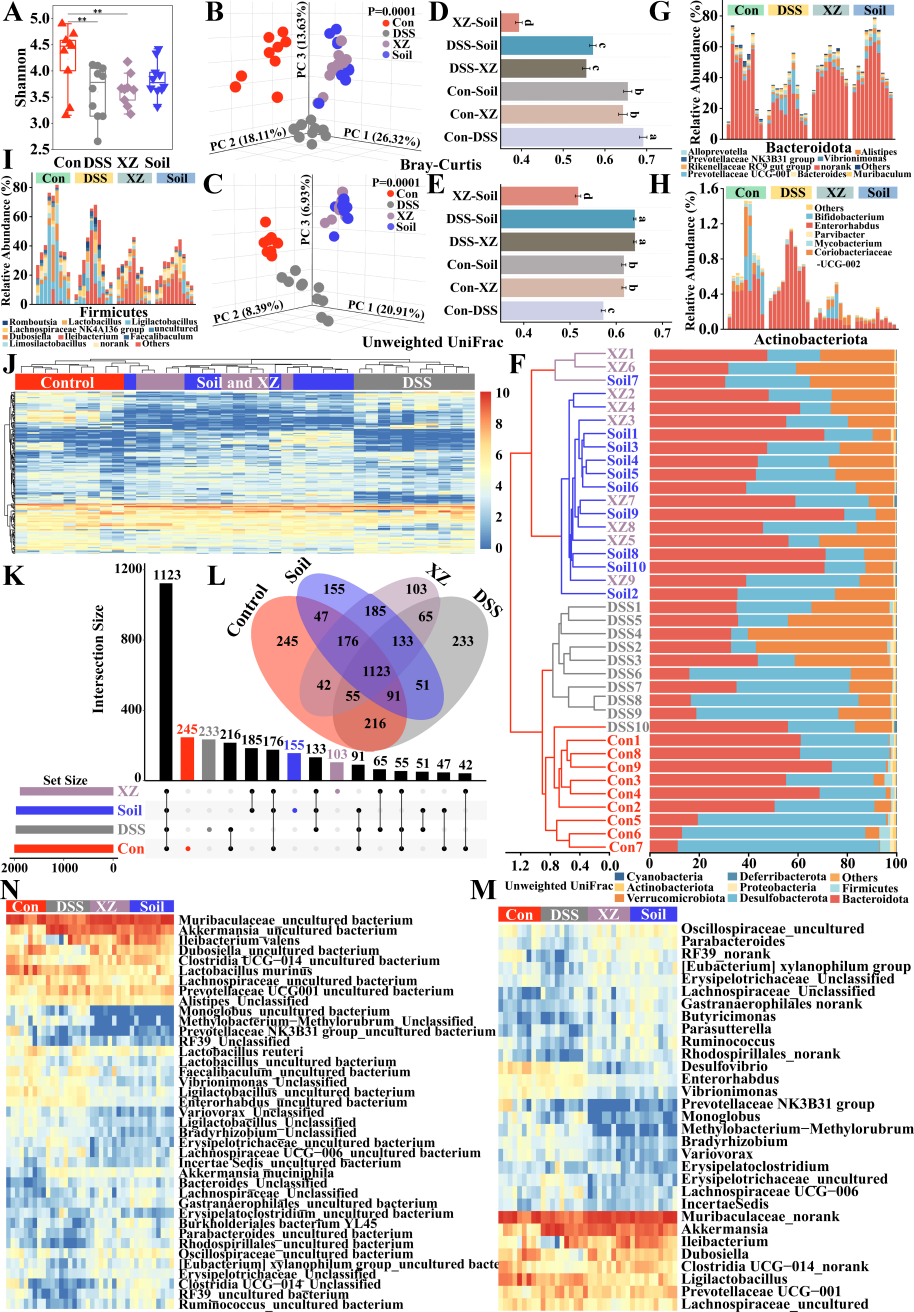
Sterile soil ingestion and LHE exposure exhibit highly similar remodeling effects on the gut microbiota in UC mice. (**A**) Shannon index. (**B and C**) PCoA based on Bray-Curtis distance and unweighted UniFrac distance, respectively. (**D and E**) Comparison of Bray-Curtis distance and unweighted UniFrac distance, respectively. (**F**) Unweighted UniFrac distance-based clustering tree (left) and relative abundance at phylum level (right). (**G–I**) Detailed relative abundance of bacterial genera within the Bacteroidota, Actinobacteriota, and Firmicutes phyla, respectively. (**J**) Hierarchical clustering heatmap of the top 200 OTUs. (**K**) Upset plot of OTUs. (**L**) Venn diagram. (**M**) Heatmap of differential bacterial genera. (**N**) Heatmap of differential bacterial species. Each bar represents the mean ± SEM. Multiple group comparisons were performed using one-way ANOVA, Kruskal-Wallis tests with Bonferroni correction. *P < 0.05, **P < 0.01, and ***P < 0.001.

Subsequently, we systematically analyzed the effects of sterile soil intake and LHE intervention on the gut microbiota of UC mice. Alpha diversity analysis revealed that the Shannon index in the Soil group showed partial recovery compared to the DSS group, reaching levels comparable to the control group without significant differences, while the XZ group still exhibited significant differences from the Control group (Fig. 3A; Table S7). PCoA based on both unweighted UniFrac distance and Bray-Curtis distance demonstrated that the Soil and XZ groups formed independent clusters with substantial overlap, while being separated from the Control and DSS groups (Fig. 3B and C). Further distance comparison analysis indicated the shortest microbial community distance between the Soil and XZ groups (Fig. 3D and E; Table S8), indicating highly similar gut microbial community structures between these two groups. UPGMA clustering analysis based on the Unweighted UniFrac distance matrix further validated this finding. The clustering tree clearly showed that samples from the Soil group and XZ group clustered together, suggesting highly convergent compositions of their gut microbiota (Fig. 3F).

At the phylum level, compared to the DSS group, both the Soil and XZ groups significantly increased the abundance of Myxococcota, with the Soil group additionally showing elevated Cyanobacteria levels and significantly reduced Actinobacteriota. The XZ group showed decreased Desulfobacterota abundance (Fig. S3A). Furthermore, the genus composition within the four major phyla—Proteobacteria, Actinobacteriota, Bacteroidota, and Firmicutes—underwent significant changes (Fig. 3G through I; Fig. S3B and Table S9).

**Fig 4:**
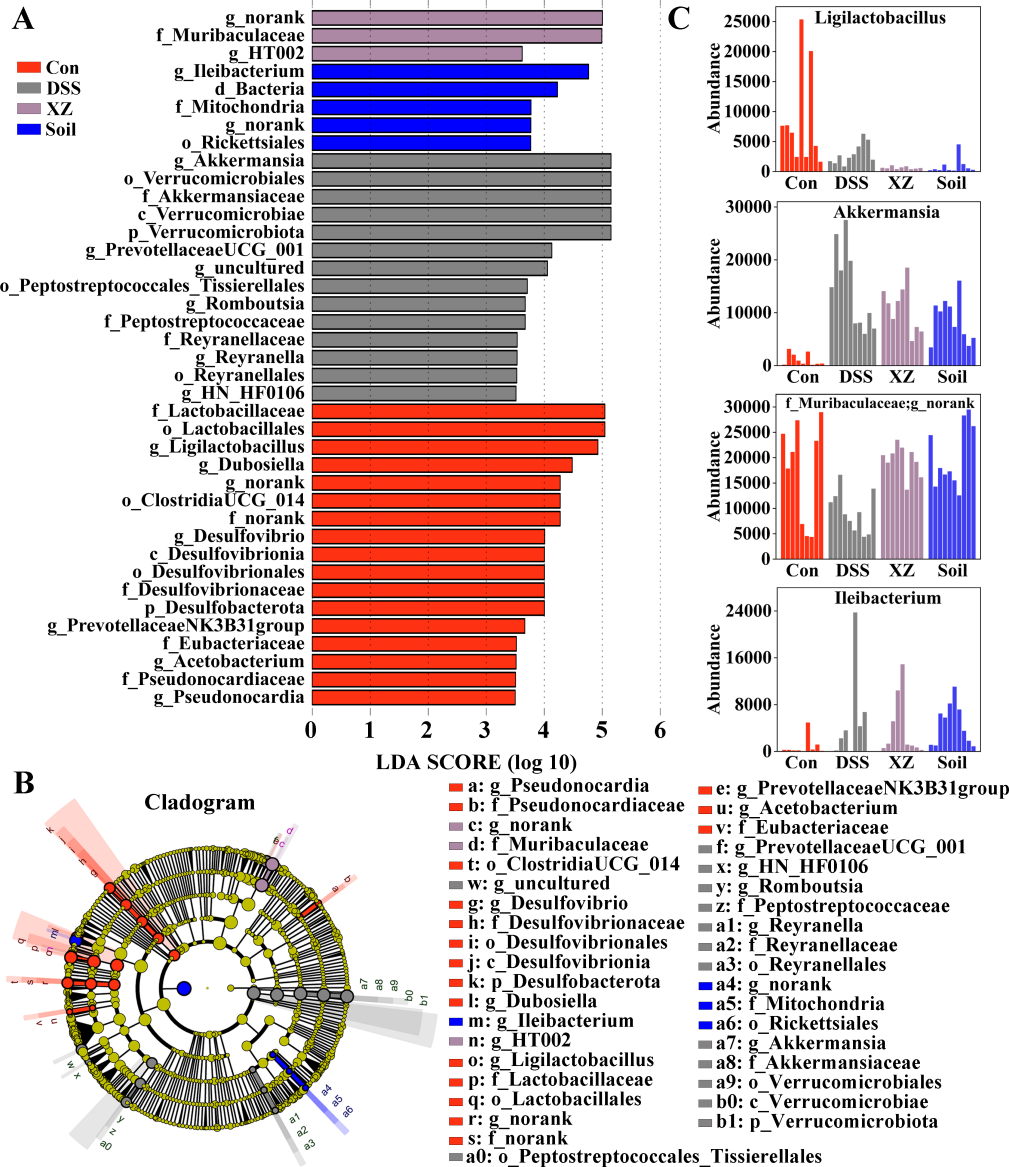
Identification of characteristic taxa based on LEfSe analysis. (**A**) Differential bacterial taxa with an LDA threshold of 3.5. (**B**) Abundance distribution of LEfSe-identified biomarkers across experimental groups. (**C**) Cladogram of intestinal microbiota.

Clustering heatmap of the top 200 OTUs revealed that the OTU profiles under Soil and XZ interventions exhibited high similarity but showed significant differences compared to the Control and DSS groups (Fig. 3J). Venn diagram analysis identified 155 OTUs unique to the Soil group and 103 OTUs unique to the XZ group (Fig. 3K and L). Furthermore, heatmap analysis of differential genera and species demonstrated that the variation patterns in abundance of specific bacterial taxa also displayed high consistency between the two intervention groups, with both effectively restoring partially lost bacterial taxa (Fig. 3M and N; Tables S10 and S11). LEfSe analysis identified significant enrichment of *HT002* and *Ileibacterium* in the XZ and Soil groups, respectively (Fig. 4A through C).

In summary, sterile soil ingestion significantly altered the gut microbiota of UC mice and induced a microbial community structure highly similar to that induced by LHE exposure. These results suggest that the simple sterile soil ingestion achieves a comparable efficacy in modulating the gut microbiota to that of the complex LHE exposure.

### Sterile soil intake and LHE exposure alleviate inflammation in colitis mice

**Fig 5:**
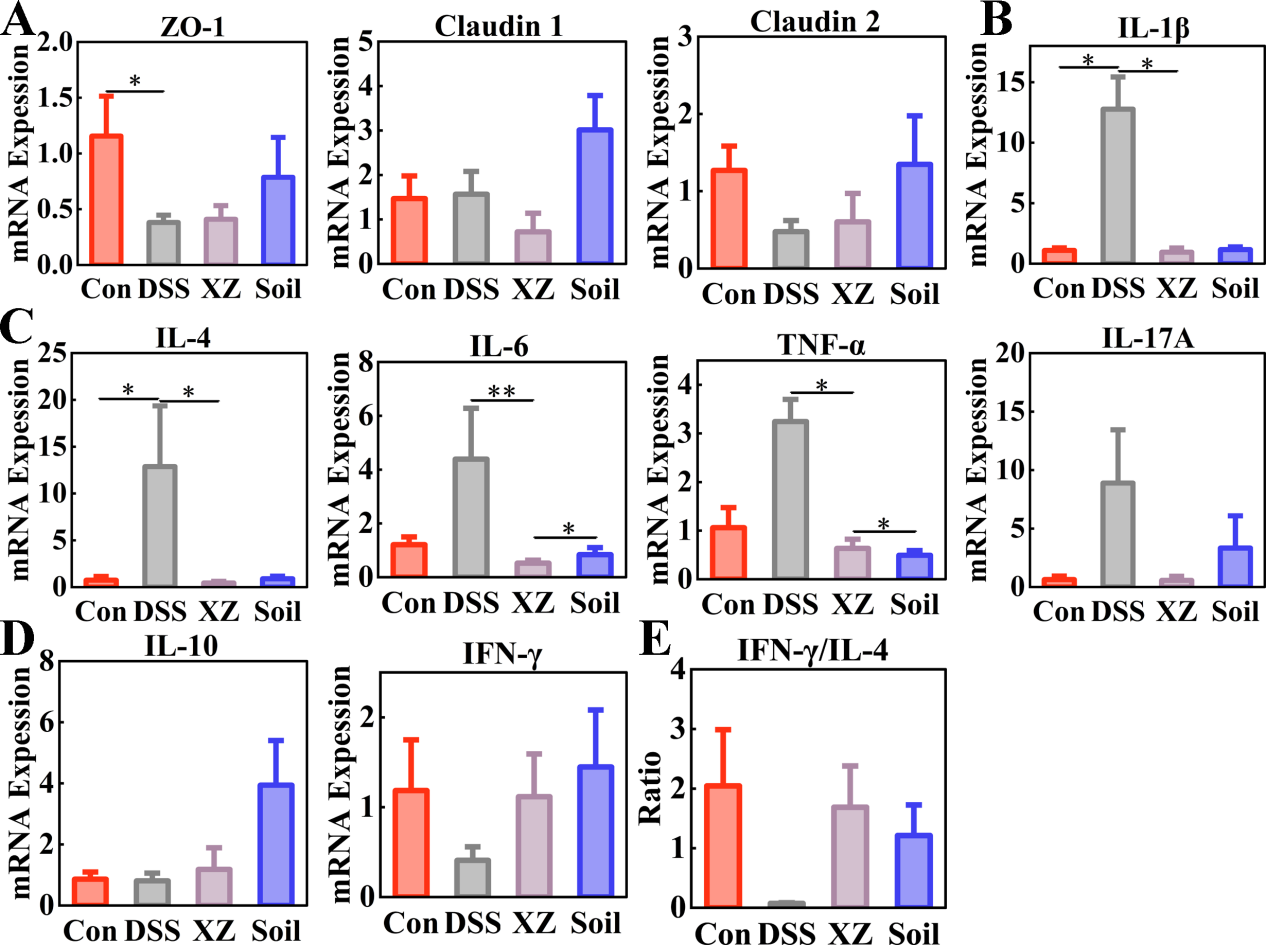
Ameliorative effects of LHE exposure and soil ingestion on inflammatory responses in UC mice. (**A**) Relative mRNA expression of tight junction genes in colon. (**B and C**) Relative mRNA expression of inflammatory cytokines. (**D**) Relative mRNA expression of anti-inflammatory cytokines. (**E**) IFN-γ/IL-4 ratio (immune balance indicator). Data are presented as mean ± SEM. Statistical significance was determined by one-way ANOVA or Kruskal-Wallis test with Bonferroni adjustment for multiple comparisons. *P < 0.05, **P < 0.01, ***P < 0.001.

The integrity of the intestinal epithelial barrier is essential for maintaining host health, ensuring efficient nutrient absorption, and stabilizing intestinal mucosal homeostasis. UC mice exhibited the downregulation of the tight junction proteins ZO-1 and Claudin 2 compared to the Control group, indicating DSS-induced damage to the intestinal mucosal barrier (Fig. 5A). Soil ingestion partially restored the levels of ZO-1, Claudin 1, and Claudin 2, suggesting its protective and restorative effects on the intestinal barrier.

Further analysis revealed that pro-inflammatory cytokines, including IL-1β, IL-4, IL-6, TNF-α, and IL-17A, were upregulated in UC mice, while the LHE and soil interventions effectively suppressed the aberrant overexpression of these cytokines, with levels trending toward those observed in the Control group (Fig. 5B and C). In addition, the anti-inflammatory cytokines IL-10 and IFN-γ were upregulated in Soil and XZ groups, indicating a shift in immune balance toward a more anti-inflammatory signaling in UC mice (Fig. 5D and E).

### Correlation between the altered microbiota induced by LHE and soil interventions and immune parameters

**Fig 6:**
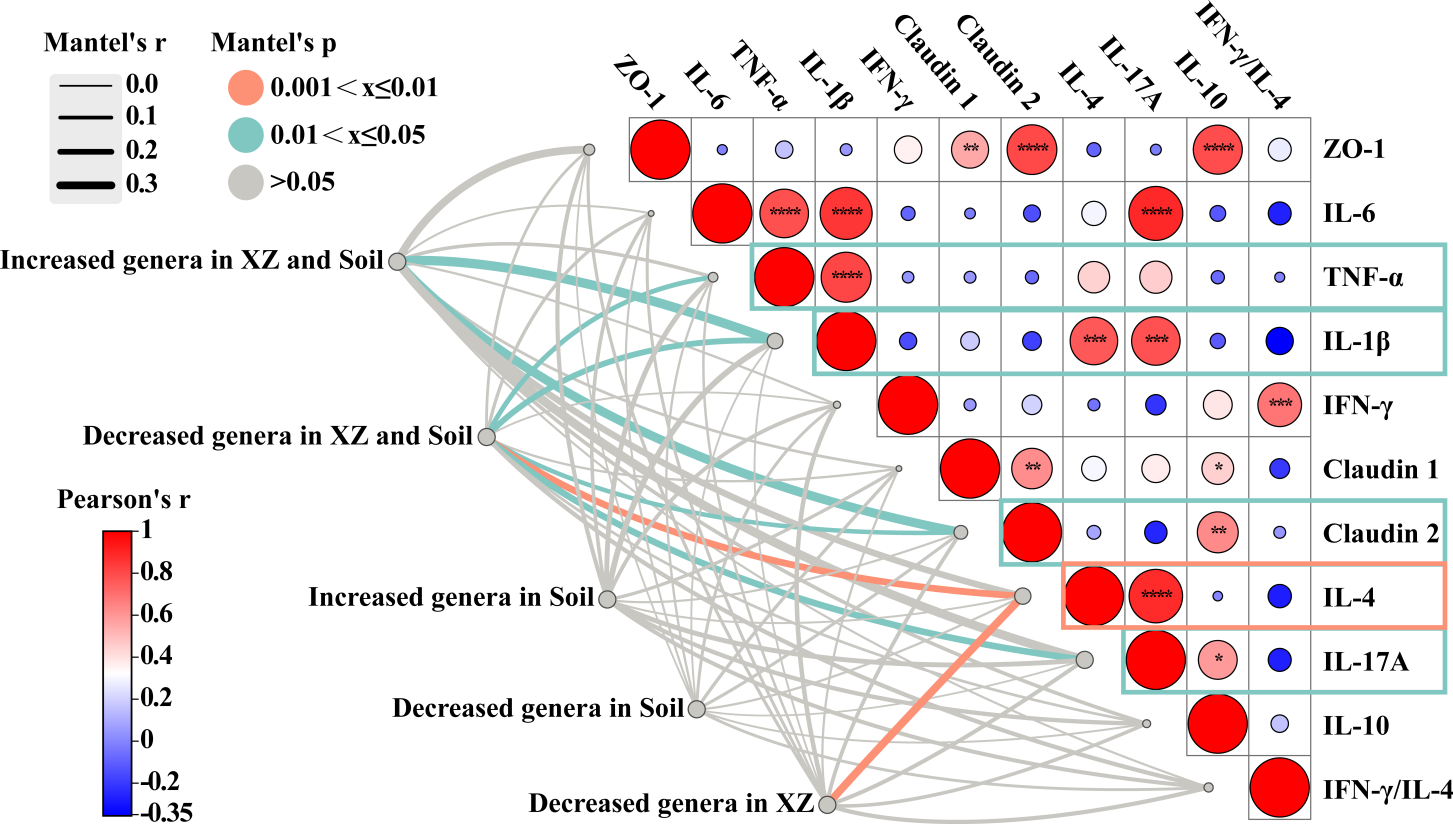
Correlation analyses among gut microbiota and immune parameters of UC mice after LCE exposure and soil intervention. The heatmap shows the results of Pearson correlation analysis of colonic cytokines. The color scale represents Pearson correlation coefficient (*P < 0.05, **P < 0.01, ***P < 0.001). The chord diagram shows the results of the Mantel test for the correlation between the gut microbiota Bray–Curtis distance matrix and immune parameters. Orange and green lines represent significant correlations, respectively (cutoff confidence level of 0.05), and the line thickness represents the value of Mantel correlation coefficient.

To further investigate the potential relationships between altered gut bacteria induced by LHE or soil interventions and host immune status, we employed the Mantel test to assess relationships between microbial composition and immune indicators (Fig. 6). Based on DSS group comparisons, differential genera were categorized by change patterns: “Increased genera in Soil and XZ groups”: Taxa significantly upregulated in both interventions, including *Muribaculaceae norank*, *Dubosiella*, *Clostridia UCG-014 norank*, *Parabacteroides*, and *Erysipelotrichaceae Unclassified*. “Decreased genera in Soil and XZ groups”: Taxa significantly downregulated in both interventions, including *Enterorhabdus*, *Vibrionimonas*, and *Lachnospiraceae UCG-006*. “Increased genera in Soil group”: *Oscillospiraceae uncultured*, *RF39 norank*, and *Ruminococcus*. “Decreased genera in Soil group”: *Ligilactobacillus*. “Decreased genera in XZ group”: *Lachnospiraceae uncultured*, *Desulfovibrio*, and *Prevotellaceae NK3B31 group*.

Mantel test results revealed significant associations between gut microbial community shifts and immune parameters. Genera co-increased in both Soil and XZ groups showed significant associations with IL-1β (*r* = 0.38, *P* = 0.025) and Claudin 2 (*r* = 0.36, *P* = 0.05). Genera co-decreased in both Soil and XZ groups showed significant associations with TNF-α (*r* = 0.16, *P* = 0.031), IL-1β (*r* = 0.21, *P* = 0.011), Claudin 2 (*r* = 0.14, *P* = 0.05), IL-4 (*r* = 0.25, *P* = 0.008), and IL-17A (*r* = 0.24, *P* = 0.016). XZ-specific decreased genera showed a significant association with IL-4 (*r* = 0.30, *P* = 0.004). However, soil-specific altered genera (whether increased or decreased) showed no significant associations with immune indicators measured.

These findings demonstrate that both LHE exposure and sterile soil intake significantly influence host immunity by modulating the abundance of specific bacterial taxa. Notably, genera whose abundance was commonly altered by both interventions exhibited strong correlations with multiple inflammatory cytokines and gut barrier indicators.

### Soil ingestion and LHE exposure increase SCFA levels

**Fig 7:**
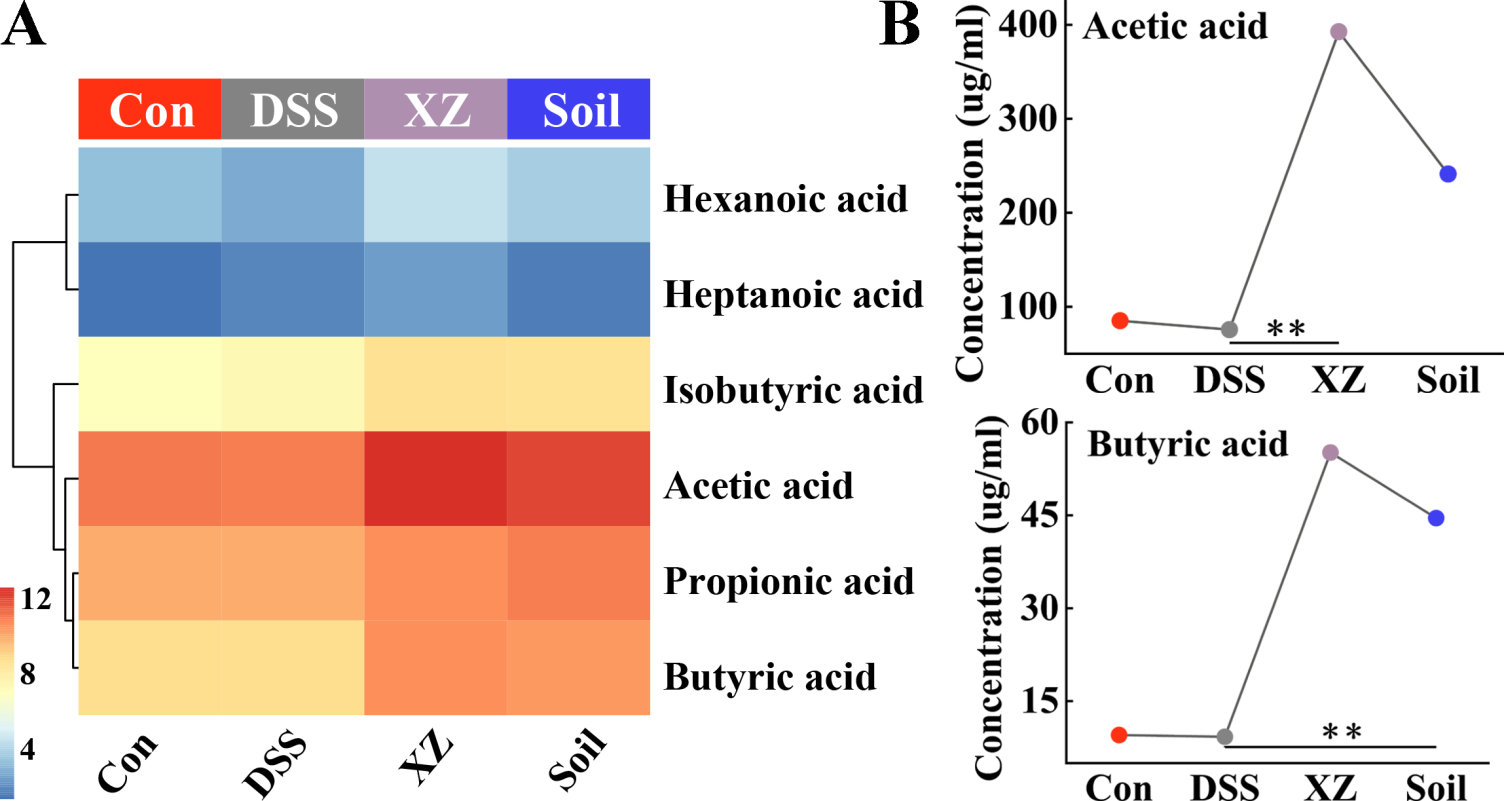
Soil ingestion and LHE exposure increase SCFA levels. (**A**) Heatmap of SCFAs. (**B**) Changes in acetic acid and butyric acid levels. Kruskal-Wallis test with Bonferroni adjustment for multiple comparisons. *P < 0.05, **P < 0.01.

To investigate the effects of low-hygiene environment exposure and soil ingestion on gut metabolism, we measured the levels of fecal SCFAs. As shown in Fig. 7A, the concentration profiles of several common SCFAs exhibited changes, with overall upregulation observed in both the Soil and XZ groups. Specifically, compared to the DSS group, LHE exposure significantly increased the concentration of acetic acid, while soil intervention significantly elevated the concentration of butyric acid (Fig. 7B). Further Mantel test correlation analysis revealed a significant association between isobutyric acid levels and IFN-γ levels (*r* = 0.30, *P* = 0.034) (Fig. S4). These results suggest that soil ingestion and LHE exposure can modulate the production of intestinal SCFAs and may potentially participate in host immune regulation processes by influencing immune factors.

## DISCUSSION

In this study, we systematically compared the modulatory effects of LHE exposure vs sterile soil intake on the gut microbiota and immune responses in DSS-induced UC mice. Both interventions effectively promoted the restoration of the intestinal ecosystem, as evidenced by the alleviation of intestinal inflammation and reshaping of the gut microbiota. Notably, the Soil and XZ groups exhibited highly similar microbial community patterns, with the Soil group showing superior outcomes in several parameters. These findings position sterile soil ingestion as an effective alternative to LHE exposure in restoring intestinal homeostasis. Given the limited accessibility to LHE exposure in urbanized societies, sterile soil intervention represents a practical therapeutic strategy for mitigating UC-related pathological disruptions.

Gut dysbiosis is a hallmark manifestation of the disrupted intestinal ecosystem in UC. In this study, we observed that DSS administration induced severe colonic damage, which was accompanied by a significant reduction in gut microbial diversity and alterations in microbial composition and structure. These findings align with previous reports (33–35). DSS treatment markedly depleted several beneficial bacterial taxa, including *Bifidobacterium pseudolongum*, *Prevotellaceae NK3B31 group*, *Parabacteroides goldsteinii*, *Lachnospiraceae UCG-001*, and *Lactobacillus murinus* (*L. murinus*). These microbes play key roles in maintaining intestinal homeostasis and suppressing inflammatory responses. For example, *L. murinus* has been reported to ameliorate UC by producing anti-inflammatory metabolites (36). Moreover, its stable colonization promoted Treg expansion, enhancing host resistance against UC inflammation (37). Furthermore, the DSS-induced gut dysbiosis was characterized by a simplification of the microbial interaction network, with reduced nodes and connections, indicating a loss of community stability. Notably, DSS primarily damages the intestinal epithelium by disrupting tight junctions, thereby impairing mucosal barrier integrity and increasing intestinal permeability (38). Consequently, gut dysbiosis may not solely act as a driver of inflammation but could, at least in part, represent a secondary consequence of the DSS-induced epithelial damage and subsequent inflammatory milieu. Collectively, the DSS-induced diversity loss, commensal depletion, and network perturbation represent a critical microbiological foundation of UC.

LHE exposure significantly alleviated intestinal inflammation in UC mice, highlighting the positive effects of moderate environmental exposures (microbes, soil) on immune regulation and intestinal health. This aligns with previous findings showing that exposure to LHE-like farms effectively maintains intestinal homeostasis and promotes immune tolerance (15, 16). Such “farm protective effects” can activate and regulate innate and adaptive immune responses, with identified beneficial exposures including contact with soil, animals, and diverse microbes (39). In our experimental setting, mice may acquire enriched environmental microbes through multiple pathways (e.g., inhalation and skin contact), thereby influencing the gut microbiota. LHE exposure also impacted intestinal metabolism in UC mice, leading to an increase in acetic acid concentration. Multiple studies have demonstrated that acetate contributes to alleviating intestinal inflammation and ameliorating pathological conditions in UC (40, 41). Besides, pro-inflammatory cytokine levels (IL-6, IL-1β, TNF-α) were significantly downregulated. These findings collectively suggest that exposure to LHE may serve as a key strategy against inflammatory disorders in the context of modern over-hygiene lifestyles.

Modern urban lifestyles, characterized by excessive hygiene and limited environmental microbial exposure, may exacerbate gut dysbiosis in inflammatory conditions. In this context, developing safe and effective alternative strategies to restore gut microbial diversity and function holds significant practical value. Zhou et al. demonstrated that sterile soil intake significantly increased gut microbial diversity and elicited beneficial immune responses (18, 42), suggesting soil as an environment-independent modulator of gut microbiota with potential for alleviating symptoms in urban populations lacking LHE exposure. Our study further supported the significant role of sterile soil in microbiota modulation and inflammation mitigation. This aligns with work by Li et al., where sterile soil restored antibiotic-disrupted microbiota and improved intestinal barrier integrity (19). Another study by Li et al. suggested that sterile soil ingestion can alleviate lung inflammation in asthmatic mice by modulating the gut microbiota (20). Besides, this study observed that soil ingestion significantly increased fecal butyric acid levels. Butyric acid can promote the repair and regeneration of intestinal epithelial cells, enhance intestinal barrier function, and exert significant protective effects against colitis by suppressing inflammatory responses (43, 44). Further analysis demonstrated that sterile soil downregulated pro-inflammatory cytokines and upregulated anti-inflammatory cytokines. Collectively, sterile soil represents a non-pharmacological, low-risk, and controllable intervention for restoring the intestinal ecosystem.

Sterile soil intake and LHE exposure demonstrated remarkable similarity in gut microbiota modulation. PCoA based on unweighted UniFrac and Bray-Curtis distances revealed substantial spatial overlap between the two groups, with the shortest intergroup distances that were significantly smaller than other groups. This observation was further validated by UPGMA hierarchical clustering analysis, which demonstrated that samples from both groups clustered closely together, forming a distinct phylogenetic branch. Besides, the Soil and LHE exposure exhibited significant overlap in multiple critical genera and species, particularly commensals associated with intestinal homeostasis maintenance and immune regulation. For instance, microbial analysis revealed increased beneficial taxa abundances, including *Parabacteroides* and *Butyricimonas* (SCFA producers with anti-inflammatory properties) (45, 46), and *Dubosiella* (an anti-inflammatory microbe enhancing host resilience) in both intervention (47). The observed similarities of gut microbiota imply that sterile soil ingestion may reshape microbial structure and function through mechanisms similar to those elicited by LHE exposure, thereby exerting anti-inflammatory effects. Collectively, these findings position sterile soil intervention as a promising alternative strategy to compensate for “microbial deprivation” in modern urban environments.

Although soils from different origins or even the same soil under varying physiological or disease contexts induce heterogeneous shifts in gut microbial taxonomic composition (18, 20, 48–50), their impacts on microbial function exhibit convergence toward beneficial outcomes. Consistently, soil interventions significantly upregulate genes involved in SCFA metabolism and essential amino acid biosynthesis (18, 19, 49), thereby driving the gut microbiota functions toward anti-inflammatory and metabolically favorable profiles. In this study, we also observed an elevation in SCFA profiles following soil intervention. These findings suggest that the ultimate health benefits of soil-based interventions primarily rely on functional rather than taxonomic reshaping.

Soil may exert regulatory effects on the gut microbiota through multiple mechanisms. First, soil contains diverse and abundant mineral components, and microorganisms possess the ability to colonize nearly all types of mineral surfaces. These minerals not only provide physical protection for microbes but also serve as essential nutrient sources to support microbial growth, metabolic activities, and functional maintenance. Through redox reactions of elements (particularly Fe and S), they supply energy to microorganisms (51). Recent studies indicate that minerals can act as signaling agents to regulate microbial adaptability and evolution (52). When soil enters the intestinal tract through dietary intake, certain minerals (e.g., smectite) with excellent adsorption properties can stably adhere to the intestinal mucus surface. By constructing biofilm microenvironments, they promote the adhesion, colonization, and proliferation of specific gut probiotics (53). Additionally, although the experimental soil samples underwent autoclave sterilization to eliminate viable microorganisms, cell wall components from inactivated bacteria (such as lipoteichoic acid) can still influence host immune responses and gut microbiota composition (54). Therefore, soil intervention may affect intestinal microbial diversity and stability through mineral-mediated nutritional support, biofilm structure formation, and immune regulation by inactivated bacterial components.

Different types of clay minerals exhibit highly specific regulatory effects on gut microbial communities. Our recent studies have demonstrated that gypsum promotes the abundance of *Lactobacillus*, *Limosilactobacillus*, and *Rikenella*, whereas halloysitum album significantly enriches *Monoglobus*. For certain bacterial taxa, such as *Faecalibaculum* and *Ileibacterium*, both gypsum and terra flava usta exhibit growth-promoting effects. Further mechanistic investigations have revealed that gut microbes can reshape microbial structure by biofilm formation on the surfaces of smectite and terra flava usta (55, 56). Soil composition analysis revealed that SiO₂ was the most abundant component (60.34%), followed by Al₂O₃ (11.73%). Notably, smectite and kaolinite with established microbial regulatory properties share SiO₂ and Al₂O₃ as their primary chemical components (57). Additionally, the soil was enriched with elements such as Ca, Zn, and Mg. Zn is an essential trace element for numerous bacteria, and its deficiency may reduce bacterial diversity and proliferation of pathogenic bacteria, resulting in gut dysbiosis (58). Ca supplementation has been shown to enhance microbial diversity and promote the colonization of beneficial bacteria (59, 60). Mg deficiency is associated with inflammation in UC, and Mg supplementation aids in the restoration of mucosal integrity (61). Moreover, clay minerals are characterized by uneven surface charge distribution, high cation exchange capacity, and large specific surface area. Under acidic conditions, their positively charged surfaces attract negatively charged probiotics (e.g., Lactobacillus and Bifidobacterium), promoting bacterial adhesion and biofilm formation, thereby enhancing probiotic colonization in the gut (57). The excellent adsorption capacity of clay minerals contributes to the removal of harmful substances, alleviating diarrhea and improving overall gut health (62). Collectively, these findings suggest that mineral components in soil may serve as key mediators in regulating gut microbiota and mitigating colitis.

While both LHE exposure and sterile soil intake demonstrate positive effects in modulating gut microecology and alleviating inflammation, sterile soil exhibits unique advantages in the context of modern urbanized lifestyles with limited environmental exposure. First, enhanced biosafety through effective pathogen elimination. Second, its flexible administration mode facilitates daily and sustainable microecological health interventions. Furthermore, standardized controllability of material source, composition, and dosage—enabling reproducible research and clinical translation. Taken together, sterile soil intervention shows promise as a simple, cost-effective, and accessible strategy for alleviating gut dysbiosis in UC.

Several limitations should be considered. First, only one type of soil collected from a specific ecological site was tested. Given that soils from different origins vary in physicochemical properties and microbial legacy effects, their impacts on gut microbiota and host health may differ substantially (49). Second, the influence of soil particle size was not investigated in this study. Soil particles of different sizes exhibit distinct physicochemical characteristics, including chemical composition, cation exchange capacity, surface reactivity, and adsorption properties, which collectively shape microbial habitats and community structure (48). Although the soil used in this experiment was ground and sieved through a 100-mesh sieve (approximately 150 μm) to ensure homogeneity, this processing method may have excluded finer particles that typically possess larger specific surface areas and tend to harbor richer microbes. Finally, this study employed only a single dose of soil supplementation. Future studies should include a comprehensive assessment of soil type, particle size, and dosage to advance these findings.

### Conclusion

In summary, although urbanization has limited individuals’ direct access to low-hygiene environments, sterile soil intake exerts comparable efficacy to environmental exposure in restoring the intestinal ecosystem in UC mice, as evidenced by the amelioration of inflammation, enhancement of barrier integrity, and favorable reshaping of the gut microbiota. Given the constraints of urban living, sterile soil intervention represents a novel and practical strategy to promote intestinal health, with promising translational potential.

## Data Availability

The raw data of 16S rRNA gene sequences were deposited in the NCBI Sequence Read Archive (SRA) database with the accession number PRJNA1367346. Other relevant data supporting the findings are available within the supplemental material.

## SUPPLEMENTAL MATERIAL

Supplemental material aem.02415-25-s0001.docx Tables S1 to S16; Fig. S1 to S4.

Graphical abstract aem.02415-25-s0002.tif Visual depiction of the study.

ASM does not own the copyrights to Supplemental Material that may be linked to, or accessed through, an article. The authors have granted ASM a non-exclusive, world-wide license to publish the Supplemental Material files. Please contact the corresponding author directly for reuse.
