# Supplementary material for: Comparable efficacy and mechanisms of sterile soil ingestion versus low hygiene exposure in DSS-induced colitis
Source: Appl Environ Microbiol. 2026 Feb 9;92(3):e02415-25. doi: 10.1128/aem.02415-25 (PMC12997849; doi:10.1128/aem.02415-25)
Supplement: Supplemental material — Tables S1 to S16; Fig. S1 to S4. [file aem.02415-25-s0001.docx]

**Supplementary material**

**Supplementary Tables**

| **Table S1. Soil composition analysis** | | | | | |
| --- | --- | --- | --- | --- | --- |
| **Compound** | **Wt%** | **Est.Error** | **Element** | **Wt%** | **Est.Error** |
| SiO_2_ | 60.34 | .24 | Si | 28.21 | .11 |
| Al_2_O_3_ | 11.73 | .16 | Al | 6.21 | .09 |
| CaO | 5.73 | .12 | Ca | 4.10 | .08 |
| Fe_2_O_3_ | 2.93 | .08 | Fe | 2.05 | .06 |
| Na_2_O | 2.26 | .07 | Na | 1.67 | .06 |
| K_2_O | 2.07 | .07 | K | 1.72 | .06 |
| MgO | 2.07 | .07 | Mg | 1.25 | .04 |
| TiO_2_ | .521 | .026 | Ti | .312 | .016 |
| P_2_O_5_ | .169 | .0084 | Px | .0735 | .0037 |
| MnO | .0539 | .0027 | Mn | .0418 | .0021 |
| SO_3_ | .0391 | .0020 | Sx | .0157 | .0008 |
| ZrO_2_ | .0264 | .0013 | Zr | .0195 | .0010 |
| SrO | .0186 | .0009 | Sr | .0157 | .0008 |
| V_2_O_5_ | .0099 | .0008 | V | .0055 | .0004 |
| CeO_2_ | .0078 | .0020 | Ce | .0063 | .0016 |
| Cr_2_O_3_ | .0068 | .0006 | Cr | .0047 | .0004 |
| Rb_2_O | .0062 | .0004 | Rb | .0056 | .0004 |
| ZnO | .0047 | .0004 | Zn | .0037 | .0003 |
| ThO_2_ | .0036 | .0018 | Th | .0031 | .0016 |
| Nb_2_O_5_ | .0035 | .0008 | Nb | .0024 | .0006 |
| Er_2_O_3_ | .0033 | .0010 | Er | .0029 | .0008 |
| NiO | .0031 | .0004 | Ni | .0025 | .0003 |
| Y2O_3_ | .0028 | .0007 | Y | .0022 | .0005 |
| CuO | .0013 | .0004 | Cu | .0011 | .0003 |

Analysis was performed using a Wavelength Dispersive X-Ray Fluorescence spectrometer (Thermo Fisher). Reporting Level is the weight more than 10 ppm and the wt% more than the Est. Error. Sum Weight% prior to normalization: 100% = 98.4%. Total Weight% Oxygen = 42.28.

| **Table S2. Primer information of RT-qPCR.** | |
| --- | --- |
| **Gene** | **Sequence (5' to 3')** |
| IL-6F | CCATAGCTACCTGGAGTACATG |
| IL-6R | TGGAAATTGGGGTAGGAAGGAC |
| TNF-αF | TGTGCTCAGAGCTTTCAACAA |
| TNF-αR | CTTGATGGTGGTGCATGAGA |
| IL-1βF | TGCCACCTTTTGACAGTGATG |
| IL-1βR | TGATGTGCTGCTGCGAGATT |
| GapdhF | ACCACAGTCCATGCCATCAC |
| GapdhR | TCCACCACCCTGTTGCTGTA |
| ZO-1F | AGCTCATAGTTCAACACAGCCTCCAG |
| ZO-1R | TTCTTCCACAGCTGAAGGACTCACAG |
| Claudin 1F | AACAACCTCTTACCCAACACCAC |
| Claudin 1R | ACAGCCAAGACCCTCATAGCC |
| Claudin 2F | CGAGAAAGAACAGCTCCGTTT |
| Claudin 2R | TTCGCTTGTCTTTTGGCTGC |
| IL10F | ATAACTGCACCCACTTCCCA |
| IL10R | TTGTCCAGCTGGTCCTTTGTT |
| IFN-γF | AACGCTACACACTGCATCTTGG |
| IFN-γR | GACTTCAAAGAGTCTGAGG |
| IL-4F | TCGGCATTTTGAACGAGGTC |
| IL-4R | GAAAAGCCCGAAAGAGTCTC |
| IL-17AF | ATCAGGACGCGCAAACATGA |
| IL-17AR | TTGGACACGCTGAGCTTTGA |

| **Table S3. Comparison of alpha diversity between DSS and Control groups** | | | |  |
| --- | --- | --- | --- | --- |
|  |  |  |  |  |
| **Alpha diversity indexes** | **Mean** | | **P-value** |  |
|  | **Control** | **DSS** |  |  |
| Observed_species | 916.33±156.89 | 864.80±135.28 | 0.447 |  |
| Chao1 | 1146.21±188.64 | 1213.71±256.36 | 0.497 |  |
| ACE | 1153.92±172.16 | 1210.17±239.69 | 0.497 |  |
| **Shannon** | 4.22±0.62 | 3.60±0.51 | **0.022** |  |
| **Simpson** | 0.93±0.07 | 0.87±0.09 | **0.013** |  |
| **Pielou_J** | 0.62±0.08 | 0.53±0.07 | **0.010** |  |
| Pd_faith | 27.96±3.37 | 25.53±2.82 | 0.095 |  |
| The P values were calculated using the *Mann–Whitney* U test. | | | |  |
|  |  |  |  |  |

| **Table S4. Major phyla of Control and DSS groups.** | | | |  |
| --- | --- | --- | --- | --- |
|  |  |  |  |  |
| **Phyla** | **Mean±SD** | | **P-value** |  |
|  | **Control** | **DSS** |  |  |
| Bacteroidota | 21512.67±11490.82 | 15108.10±5853.99 | 0.211 |  |
| Firmicutes | 22272.33±10776.91 | 16184.70±10741.68 | 0.182 |  |
| Firmicutes / Bacteroidota | 2.23±2.66 | 1.53±1.58 | 0.604 |  |
| **Verrucomicrobiota** | 1109.22±1171.85 | 14381.50±7818.34 | **0.000** |  |
| **Desulfobacterota** | 932.33±681.03 | 278.00±194.33 | **0.006** |  |
| Proteobacteria | 474.11±216.92 | 362.20±207.01 | 0.211 |  |
| Actinobacteriota | 379.89±193.78 | 314.30±134.99 | 0.497 |  |
| Cyanobacteria | 8.33±5.68 | 19.30±15.60 | 0.079 |  |
| Deferribacterota | 8.33±9.43 | 49.90±120.94 | 0.211 |  |
| Myxococcota | 7.78±10.22 | 9.00±7.51 | 0.447 |  |
| The P values were calculated using the *Mann–Whitney* U test. | | | |  |
|  |  |  |  |  |

| **Table S5. Top 20 most abundant genera in Control and DSS groups.** | | | |  |
| --- | --- | --- | --- | --- |
|  |  |  |  |  |
| **Genera** | **Mean±SD** | | **P-value** |  |
|  | **Control** | **DSS** |  |  |
| Muribaculaceae_norank | 17662.33±9876.80 | 9436.80±4057.23 | 0.133 |  |
| **Akkermansia** | 1108.56±1171.21 | 14381.30±7818.40 | **0.000** |  |
| **Ligilactobacillus** | 8652.22±8386.86 | 2939.30±1766.01 | **0.043** |  |
| Ileibacterium | 807.67±1579.03 | 4071.50±7287.65 | 1.000 |  |
| **Dubosiella** | 3503.22±3631.48 | 253.90±271.57 | **0.001** |  |
| **Prevotellaceae UCG-001** | 523.78±421.82 | 2664.10±2218.73 | **0.001** |  |
| **Clostridia UCG-014_norank** | 1832.67±1785.15 | 153.30±58.21 | **0.013** |  |
| Lachnospiraceae_uncultured | 673.33±543.83 | 1167.70±1181.11 | 0.400 |  |
| **Desulfovibrio** | 925.44±679.55 | 247.30±195.73 | **0.001** |  |
| Enterorhabdus | 197.78±79.40 | 298.10±134.28 | 0.211 |  |
| **Prevotellaceae NK3B31 group** | 382.67±545.60 | 7.10±8.10 | **0.017** |  |
| Vibrionimonas | 193.22±184.43 | 145.60±76.96 | 0.968 |  |
| Lachnospiraceae UCG-006 | 46.56±59.31 | 188.60±217.44 | 0.065 |  |
| Oscillospiraceae_uncultured | 139.89±138.05 | 66.90±88.85 | 0.182 |  |
| Monoglobus | 95.11±115.81 | 73.40±123.51 | 0.243 |  |
| **RF39_norank** | 144.67±129.99 | 26.70±41.74 | **0.043** |  |
| Erysipelotrichaceae_uncultured | 31.44±11.84 | 119.20±137.30 | 0.133 |  |
| Methylobacterium-Methylorubrum | 80.00±87.53 | 62.70±58.52 | 0.842 |  |
| IncertaeSedis | 23.44±21.69 | 108.90±190.14 | 0.243 |  |
| Variovorax | 69.89±54.92 | 64.20±40.25 | 0.905 |  |
| The P values were calculated using the *Mann–Whitney* U test. | | | |  |
|  |  |  |  |  |

| **Table S6. Top 20 most abundant species in Control and DSS groups.** | | | |  |
| --- | --- | --- | --- | --- |
|  |  |  |  |  |
| **Species** | **Mean** | | **P-value** |  |
|  | **Control** | **DSS** |  |  |
| Muribaculaceae_uncultured bacterium | 17662.33±9876.80 | 9436.80±4057.23 | 0.133 |  |
| **Akkermansia_uncultured bacterium** | 1093.33±1156.02 | 14189.00±7709.67 | **0.000** |  |
| **Lactobacillus murinus** | 8025.78±7847.25 | 2701.80±1654.72 | **0.043** |  |
| Ileibacterium valens | 807.67±1579.03 | 4071.50±7287.65 | 1.00 |  |
| **Dubosiella_uncultured bacterium** | 3503.22±3631.48 | 253.90±271.57 | **0.001** |  |
| **Prevotellaceae UCG-001_uncultured bacterium** | 523.78±421.82 | 2664.10±2218.73 | **0.001** |  |
| Lachnospiraceae NK4A136 group_uncultured bacterium | 770.44±630.94 | 1576.00±1802.42 | 1.000 |  |
| Limosilactobacillus_uncultured bacterium | 1415.11±1475.59 | 804.80±968.55 | 0.182 |  |
| Lactobacillus_Unclassified | 1373.11±1430.22 | 826.80±1087.52 | 0.133 |  |
| Lachnospiraceae_uncultured bacterium | 671.78±543.30 | 1166.70±1180.87 | 0.400 |  |
| **Clostridia UCG-014_uncultured bacterium** | 1637.78±1602.95 | 137.20±66.08 | **0.008** |  |
| Alloprevotella_uncultured bacterium | 572.44±287.27 | 648.70±496.98 | 0.780 |  |
| **Desulfovibrio_uncultured bacterium** | 925.22±679.36 | 247.20±195.82 | **0.001** |  |
| Bacteroides_uncultured bacterium | 629.67±495.14 | 465.90±222.89 | 0.447 |  |
| Lactobacillus reuteri | 582.78±586.60 | 293.70±362.78 | 0.133 |  |
| Alistipes_uncultured bacterium | 473.78±281.34 | 371.30±352.69 | 0.447 |  |
| **Alistipes_Unclassified** | 270.56±234.79 | 505.60±286.85 | **0.010** |  |
| Muribaculum intestinale | 248.56±154.80 | 463.90±300.03 | 0.133 |  |
| **Ligilactobacillus_uncultured bacterium** | 531.11±465.68 | 200.20±103.32 | **0.035** |  |
| Romboutsia_uncultured bacterium | 16.89±13.37 | 620.70±829.44 | 0.156 |  |
| The P values were calculated using the *Mann–Whitney* U test. | | | |  |
|  |  |  |  |  |

| **Table S7. Comparison of alpha diversity among the Control, DSS, XZ, and Soil groups.** | | | | | | | | | | |
| --- | --- | --- | --- | --- | --- | --- | --- | --- | --- | --- |
| **Alpha diversity indexes** | **Mean** | | | | **Significance** | | | | | |
|  | **Con** | **DSS** | **XZ** | **Soil** | **Con-DSS** | **Con-XZ** | **Con-Soil** | **DSS-XZ** | **DSS-Soil** | **XZ-Soil** |
| Observed_species | 916.33±156.89 | 864.80±135.28 | 872.33±131.02 | 947.5±120.12 | —— | —— | —— | —— | —— | —— |
| Chao1 | 1146.21±188.64 | 1213.71±256.36 | 1211.23±212.84 | 1201.±153.46 | —— | —— | —— | —— | —— | —— |
| ACE | 1153.92±172.16 | 1210.17±239.69 | 1206.68±166.26 | 1216.±149.03 | —— | —— | —— | —— | —— | —— |
| **Shannon** | 4.22±0.62 | 3.60±0.51 | 3.63±0.30 | 3.85±0.34 | **※※** | **※※** | —— | —— | —— | —— |
| Simpson | 0.93±0.07 | 0.87±0.09 | 0.91±0.03 | 0.92±0.02 | —— | —— | —— | —— | —— | —— |
| Pielou_J | 0.62±0.08 | 0.53±0.07 | 0.54±0.03 | 0.56±0.04 | —— | —— | —— | —— | —— | —— |
| Pd_faith | 27.96±3.37 | 25.53±2.82 | 25.12±3.16 | 26.78±2.87 | —— | —— | —— | —— | —— | —— |
| For homoscedastic data, one-way ANOVA followed by LSD post hoc test was employed;  For heteroscedastic data, Kruskal-Wallis test with Bonferroni correction was used. ^※^*P* < 0.05, ^※※^*P* < 0.01, ^※※※^*P* < 0.01. | | | | | | | | | | |

| **Table S8A. Comparison of Unweighted UniFrac distances among Control, DSS, XZ and Soil groups** | | | | | | |
| --- | --- | --- | --- | --- | --- | --- |
|  | **Con-DSS** | **Con-XZ** | **Con-Soil** | **DSS-XZ** | **DSS-Soil** | **XZ-Soil** |
| **Con-XZ** | 1.00 | **0.00** | 0.38 | **0.00** | **0.00** | **0.00** |
| **Con-DSS** |  | 1.00 | 0.12 | **0.00** | **0.00** | **0.00** |
| **Con-Soil** |  |  | 1.00 | **0.00** | **0.00** | **0.00** |
| **DSS-XZ** |  |  |  | 1.00 | 0.20 | **0.00** |
| **DSS-Soil** |  |  |  |  | 1.00 | **0.00** |
| **XZ-Soil** |  |  |  |  |  | 1.00 |
| **Table S8B. Comparison of Bray-Curtis distances among Control, DSS, XZ and Soil groups** | | | | | | |
|  | **Con-XZ** | **Con-DSS** | **Con-Soil** | **DSS-XZ** | **DSS-Soil** | **XZ-Soil** |
| **Con-XZ** | 1.00 | **0.00** | 1.00 | **0.00** | **0.00** | **0.00** |
| **Con-DSS** |  | 1.00 | **0.00** | **0.00** | **0.00** | 0.11 |
| **Con-Soil** |  |  | 1.00 | **0.01** | **0.00** | **0.00** |
| **DSS-XZ** |  |  |  | 1.00 | 1.00 | **0.00** |
| **DSS-Soil** |  |  |  |  | 1.00 | **0.00** |
| **XZ-Soil** |  |  |  |  |  | 1.00 |
| For homoscedastic data, one-way ANOVA followed by LSD post hoc test was employed;  For heteroscedastic data, Kruskal-Wallis test with Bonferroni correction was used. | | | | | | |

| **Table S9. Major phyla of Control, DSS, XZ and Soil groups.** | | | | | | | | | | |
| --- | --- | --- | --- | --- | --- | --- | --- | --- | --- | --- |
| **Phyla** | **Mean** | | | | **Significance** | | | | | |
|  | **Con** | **DSS** | **XZ** | **Soil** | **Con-DSS** | **Con-XZ** | **Con-Soil** | **DSS-XZ** | **DSS-Soil** | **XZ-Soil** |
| Bacteroidota | 21512.67±11490.82 | 15108.10±5853.99 | 23052.78±4486.27 | 24817.50±8312.21 | —— | —— | —— | —— | —— | —— |
| Firmicutes | 22272.33±10776.91 | 16184.70±10741.68 | 12356.22±5082.80 | 12772.40±5028.40 | —— | —— | —— | —— | —— | —— |
| Firmicutes/Bacteroidota | 2.23±2.66 | 1.53±1.58 | 0.58±0.32 | 0.63±0.40 | —— | —— | —— | —— | —— | —— |
| **Verrucomicrobiota** | 1109.22±1171.85 | 14381.50±7818.34 | 10885.00±4461.37 | 8646.00±4164.55 | **※※※** | **※※※** | **※** | —— | —— | —— |
| **Desulfobacterota** | 932.33±681.03 | 278.00±194.33 | 23.44±8.13 | 74.40±85.17 | —— | **※※※** | **※※** | **※※** | —— | —— |
| **Proteobacteria** | 474.11±216.92 | 362.20±207.01 | 164.56±53.88 | 194.10±130.75 | —— | **※※** | **※** | —— | —— | —— |
| **Actinobacteriota** | 379.89±193.78 | 314.30±134.99 | 125.33±64.40 | 61.70±23.92 | —— | **※** | **※※※** | —— | **※※※** | —— |
| **Cyanobacteria** | 8.33±5.68 | 19.30±15.60 | 32.78±26.99 | 112.50±131.00 | —— | —— | **※※※** | —— | **※** | —— |
| Deferribacterota | 8.33±9.43 | 49.90±120.94 | 67.11±125.16 | 29.50±41.39 | —— | —— | —— | —— | —— | —— |
| **Myxococcota** | 7.78±10.22 | 9.00±7.51 | 1.33±2.69 | 0.30±0.67 | —— | —— | **※※** | **※※** | **※※※** | —— |
| ^※^*P* < 0.05, ^※※^*P* < 0.01, ^※※※^*P* < 0.01. The P values were caculated using the Kruskal-Wallis test with Bonferroni correction. | | | | | | | | | | |

| **Table S10. Top 20 most abundant genera in the Control, DSS, XZ, and Soil groups.** | | | | | | | | | | |
| --- | --- | --- | --- | --- | --- | --- | --- | --- | --- | --- |
| **Genera** | **Mean** | | | | **Significance** | | | | | |
|  | **Con** | **DSS** | **XZ** | **Soil** | **Con-DSS** | **Con-XZ** | **Con-Soil** | **DSS-XZ** | **DSS-Soil** | **XZ-Soil** |
| **Muribaculaceae_norank** | 17662.33±9876.80 | 9436.80±4057.23 | 19523.56±3022.96 | 20254.10±6209.46 | —— | —— | —— | **※** | **※※** | —— |
| **Akkermansia** | 1108.56±1171.21 | 14381.30±7818.40 | 10885.00±4461.37 | 8646.00±4164.55 | **※※※** | **※※※** | **※** | —— | —— | —— |
| **Ileibacterium** | 807.67±1579.03 | 4071.50±7287.65 | 3920.00±5267.72 | 4682.40±3553.28 | —— | —— | **※** | —— | —— | —— |
| **Ligilactobacillus** | 8652.22±8386.86 | 2939.30±1766.01 | 608.44±216.13 | 876.00±1332.62 | —— | **※※※** | **※※※** | —— | **※** | —— |
| **Dubosiella** | 3503.22±3631.48 | 253.90±271.57 | 2834.44±2729.04 | 1231.70±549.27 | **※※※** | —— | —— | **※※** | **※** | —— |
| **Prevotellaceae UCG-001** | 523.78±421.82 | 2664.10±2218.73 | 782.11±453.13 | 1499.70±812.67 | **※※** | —— | **※** | —— | —— | —— |
| **Clostridia UCG-014_norank** | 1832.67±1785.15 | 153.30±58.21 | 1155.67±1250.10 | 1469.70±1062.76 | **※** | —— | —— | **※** | **※※** | —— |
| **Lachnospiraceae_uncultured** | 673.33±543.83 | 1167.70±1181.11 | 245.00±170.85 | 537.50±533.91 | —— | —— | —— | **※** | —— | —— |
| **Desulfovibrio** | 925.44±679.55 | 247.30±195.73 | 7.11±4.65 | 33.40±37.32 | —— | **※※※** | **※※※** | **※※※** | —— | —— |
| **Enterorhabdus** | 197.78±79.40 | 298.10±134.28 | 50.89±19.02 | 43.50±19.14 | —— | **※** | **※※** | **※※※** | **※※※** | —— |
| **Oscillospiraceae_uncultured** | 139.89±138.05 | 66.90±88.85 | 146.00±128.80 | 229.90±148.75 | —— | —— | —— | —— | **※** | —— |
| **Vibrionimonas** | 193.22±184.43 | 145.60±76.96 | 23.11±20.26 | 24.30±33.30 | —— | **※※** | **※※** | **※※** | **※※※** | —— |
| **Prevotellaceae NK3B31 group** | 382.67±545.60 | 7.10±8.10 | 0.00±0.00 | 3.30±5.42 | —— | **※※※** | —— | **※** | —— | —— |
| **RF39_norank** | 144.67±129.99 | 26.70±41.74 | 78.67±84.42 | 117.00±99.88 | —— | —— | —— | —— | **※** | —— |
| **Parabacteroides** | 52.89±50.79 | 17.60±12.99 | 146.22±174.06 | 110.40±86.95 | —— | —— | —— | **※※** | **※※** | —— |
| **Lachnospiraceae UCG-006** | 46.56±59.31 | 188.60±217.44 | 13.56±16.79 | 21.20±22.97 | —— | —— | —— | **※※** | **※** | —— |
| **[Eubacterium] xylanophilum group** | 60.33±66.43 | 15.00±19.38 | 84.11±90.16 | 107.50±143.04 | —— | —— | —— | **※** | **※** | —— |
| **Erysipelotrichaceae_Unclassified** | 66.56±50.27 | 16.80±10.36 | 77.11±48.45 | 51.00±14.18 | **※※** | —— | —— | **※※※** | **※※** | —— |
| **Ruminococcus** | 43.44±36.79 | 22.70±44.59 | 29.11±24.15 | 106.50±85.47 | —— | —— | —— | —— | **※** | —— |
| Parasutterella | 17.44±20.80 | 17.50±19.38 | 61.11±52.34 | 93.60±103.36 | —— | —— | —— | —— | —— | —— |
| ^※^*P* < 0.05, ^※※^*P* < 0.01, ^※※※^*P* < 0.01. The P values were calculated using the Kruskal-Wallis test with Bonferroni correction. | | | | | | | | | | |

| **Table S11. Top 20 most abundant species in the Control, DSS, XZ, and Soil groups.** | | | | | | | | | | |
| --- | --- | --- | --- | --- | --- | --- | --- | --- | --- | --- |
| **Species** | **Mean** | | | | **Significance** | | | | | |
|  | **Con** | **DSS** | **XZ** | **Soil** | **Con-DSS** | **Con-XZ** | **Con-Soil** | **DSS-XZ** | **DSS-Soil** | **XZ-Soil** |
| **Muribaculaceae_uncultured bacterium** | 17662.33±9876.80 | 9436.80±4057.23 | 19523.56±3022.96 | 20254.10±6209.46 | —— | —— | —— | **※** | **※※** | —— |
| **Akkermansia_uncultured bacterium** | 1093.33±1156.02 | 14189.00±7709.67 | 10769.11±4392.77 | 8533.10±4097.99 | **※※※** | **※※※** | **※** | —— | —— | —— |
| **Ileibacterium valens** | 807.67±1579.03 | 4071.50±7287.65 | 3920.00±5267.72 | 4682.40±3553.28 | —— | —— | **※** | —— | —— | —— |
| **Lactobacillus murinus** | 8025.78±7847.25 | 2701.80±1654.72 | 535.44±197.30 | 796.60±1250.27 | —— | **※※※** | **※※※** | **※** | **※** | —— |
| **Dubosiella_uncultured bacterium** | 3503.22±3631.48 | 253.90±271.57 | 2834.44±2729.04 | 1231.70±549.27 | **※※※** | —— | —— | **※※** | **※** | —— |
| **Prevotellaceae UCG-001_uncultured bacterium** | 523.78±421.82 | 2664.10±2218.73 | 782.11±453.13 | 1499.70±812.67 | **※※** | —— | **※** | —— | —— | —— |
| **Clostridia UCG-014_uncultured bacterium** | 1637.78±1602.95 | 137.20±66.08 | 1077.44±1154.67 | 1352.80±980.46 | **※※** | —— | —— | **※** | **※※** | —— |
| Lachnospiraceae NK4A136 group_uncultured bacterium | 770.44±630.94 | 1576.00±1802.42 | 588.33±945.77 | 1087.10±1645.80 | —— | —— | —— | —— | —— | —— |
| Lactobacillus_Unclassified | 1373.11±1430.22 | 826.80±1087.52 | 437.78±290.43 | 321.30±392.37 | —— | —— | —— | —— | —— | —— |
| Limosilactobacillus_uncultured bacterium | 1415.11±1475.59 | 804.80±968.55 | 413.00±237.30 | 310.30±370.79 | —— | —— | —— | —— | —— | —— |
| **Lachnospiraceae_uncultured bacterium** | 671.78±543.30 | 1166.70±1180.87 | 244.44±170.17 | 534.90±530.54 | —— | —— | —— | **※** | —— | —— |
| Alloprevotella_uncultured bacterium | 572.44±287.27 | 648.70±496.98 | 691.33±381.86 | 723.40±572.81 | —— | —— | —— | —— | —— | —— |
| Bacteroides_uncultured bacterium | 629.67±495.14 | 465.90±222.89 | 319.67±255.89 | 471.10±401.00 | —— | —— | —— | —— | —— | —— |
| **Alistipes_Unclassified** | 270.56±234.79 | 505.60±286.85 | 547.44±280.77 | 467.40±271.82 | —— | **※** | —— | —— | —— | —— |
| Alistipes_uncultured bacterium | 473.78±281.34 | 371.30±352.69 | 401.00±377.39 | 478.90±249.94 | —— | —— | —— | —— | —— | —— |
| Muribaculum intestinale | 248.56±154.80 | 463.90±300.03 | 222.67±73.94 | 294.50±126.46 | —— | —— | —— | —— | —— | —— |
| Lactobacillus reuteri | 582.78±586.60 | 293.70±362.78 | 199.89±123.91 | 168.90±164.03 | —— | —— | —— | —— | —— | —— |
| **Desulfovibrio_uncultured bacterium** | 925.22±679.36 | 247.20±195.82 | 7.11±4.65 | 33.40±37.32 | —— | **※※※** | **※※※** | **※※※** | —— | —— |
| **Faecalibaculum_uncultured bacterium** | 323.67±204.24 | 270.40±273.03 | 274.22±433.57 | 39.20±35.30 | —— | —— | **※** | —— | —— | —— |
| **Ligilactobacillus_uncultured bacterium** | 531.11±465.68 | 200.20±103.32 | 59.33±24.71 | 64.10±75.10 | —— | **※※※** | **※※※** | —— | **※** | —— |
| ^※^*P* < 0.05, ^※※^*P* < 0.01, ^※※※^*P* < 0.01. The P values were calculated using the Kruskal-Wallis test with Bonferroni correction. | | | | | | | | | | |

| **Table S12. Comparison of alpha diversity among the control, DSS, and XZ groups.** | | | | | | |
| --- | --- | --- | --- | --- | --- | --- |
|  |  |  |  |  |  |  |
| **Alpha diversity indexes** | **Mean** | | | **Significance** | | |
|  | **Con** | **DSS** | **XZ** | **Con-DSS** | **Con-XZ** | **DSS-XZ** |
| Observed_species | 916.33±156.89 | 864.80±135.28 | 872.33±131.02 | —— | —— | —— |
| Chao1 | 1146.21±188.64 | 1213.71±256.36 | 1211.23±212.84 | —— | —— | —— |
| ACE | 1153.92±172.16 | 1210.17±239.69 | 1206.68±166.26 | —— | —— | —— |
| **Shannon** | 4.22±0.62 | 3.60±0.51 | 3.63±0.30 | **※** | **※** | —— |
| Simpson | 0.93±0.07 | 0.87±0.09 | 0.91±0.03 | —— | —— | —— |
| **Pielou_J** | 0.62±0.08 | 0.53±0.07 | 0.54±0.03 | **※※** | **※※** | —— |
| Pd_faith | 27.96±3.37 | 25.53±2.82 | 25.12±3.16 | —— | —— | —— |
| ^※^*P* < 0.05, ^※※^*P* < 0.01. Group differences were assessed by one-way ANOVA with LSD post hoc test. | | | | | | |
|  |  |  |  |  |  |  |

| **Table S13A. Comparison of Unweighted UniFrac distances among Control, DSS, and XZ groups** | | | |
| --- | --- | --- | --- |
|  |  |  |  |
|  |  |  |  |
|  | **Con-XZ** | **Con-DSS** | **DSS-XZ** |
| **Con-XZ** | 1 | **0.00** | **0.00** |
| **Con-DSS** |  | 1 | **0.00** |
| **DSS-XZ** |  |  | 1 |
| **Table S13B. Comparison of Bray-Curtis distances among Control, DSS, and XZ groups** | | | |
|  |  |  |  |
|  |  |  |  |
|  | **Con-XZ** | **Con-DSS** | **DSS-XZ** |
| **Con-XZ** | 1 | **0.00** | **0.00** |
| **Con-DSS** |  | 1 | **0.00** |
| **DSS-XZ** |  |  | 1 |
| For homoscedastic data, one-way ANOVA followed by LSD post hoc test was employed;  For heteroscedastic data, Kruskal-Wallis test with Bonferroni correction was used. | | | |

| **Table S14. Major phyla of Control, DSS and XZ groups.** | | | | | | |
| --- | --- | --- | --- | --- | --- | --- |
|  |  |  |  |  |  |  |
| **Phyla** | **Mean** | | | **Significance** | | |
|  | **Con** | **DSS** | **XZ** | **Con-DSS** | **Con-XZ** | **DSS-XZ** |
| Bacteroidota | 21512.67±11490.82 | 15108.10±5853.99 | 23052.78±4486.27 | —— | —— | —— |
| Firmicutes | 22272.33±10776.91 | 16184.70±10741.68 | 12356.22±5082.80 | —— | —— | —— |
| Firmicutes/Bacteroidota | 2.23±2.66 | 1.53±1.58 | 0.58±0.32 | —— | —— | —— |
| **Verrucomicrobiota** | 1109.22±1171.85 | 14381.50±7818.34 | 10885.00±4461.37 | **※※※** | **※※** | —— |
| **Desulfobacterota** | 932.33±681.03 | 278.00±194.33 | 23.44±8.13 | **※※※** | —— | **※** |
| **Proteobacteria** | 474.11±216.92 | 362.20±207.01 | 164.56±53.88 | —— | **※※※** | **※** |
| **Actinobacteriota** | 379.89±193.78 | 314.30±134.99 | 125.33±64.40 | —— | **※※** | **※** |
| **Cyanobacteria** | 8.33±5.68 | 19.30±15.60 | 32.78±26.99 | —— | **※※** | —— |
| Deferribacterota | 8.33±9.43 | 49.90±120.94 | 67.11±125.16 | —— | —— | —— |
| Myxococcota | 7.78±10.22 | 9.00±7.51 | 1.33±2.69 | —— | —— | **※※** |
| ^※^*P* < 0.05, ^※※^*P* < 0.01, ^※※※^*P* < 0.01. The P values were caculated using the Kruskal-Wallis test with Bonferroni correction. | | | | | | |
|  |  |  |  |  |  |  |

| **Table S15. Top 20 most abundant genera in the Control, DSS and XZ groups.** | | | | | | |
| --- | --- | --- | --- | --- | --- | --- |
|  |  |  |  |  |  |  |
| **Genera** | **Mean** | | | **Significance** | | |
|  | **Con** | **DSS** | **XZ** | **Con-DSS** | **Con-XZ** | **DSS-XZ** |
| **Muribaculaceae_norank** | 17662.33±9876.80 | 9436.80±4057.23 | 19523.56±3022.96 | —— | —— | **※** |
| **Akkermansia** | 1108.56±1171.21 | 14381.30±7818.40 | 10885.00±4461.37 | **※※※** | **※※** | —— |
| **Ligilactobacillus** | 8652.22±8386.86 | 2939.30±1766.01 | 608.44±216.13 | —— | **※※※** | **※※** |
| Ileibacterium | 807.67±1579.03 | 4071.50±7287.65 | 3920.00±5267.72 | —— | —— | —— |
| **Dubosiella** | 3503.22±3631.48 | 253.90±271.57 | 2834.44±2729.04 | **※※** | —— | **※※** |
| Limosilactobacillus | 2195.22±2165.56 | 1304.10±1569.97 | 786.44±414.52 | —— | —— | —— |
| **Prevotellaceae UCG-001** | 523.78±421.82 | 2664.10±2218.73 | 782.11±453.13 | **※※** | —— | —— |
| **Clostridia UCG-014_norank** | 1832.67±1785.15 | 153.30±58.21 | 1155.67±1250.10 | **※※** | —— | **※** |
| Lachnospiraceae NK4A136 group | 772.00±630.55 | 1576.30±1802.69 | 589.67±945.87 | —— | —— | —— |
| Lactobacillus | 1533.22±1541.07 | 900.50±1172.17 | 479.67±310.94 | —— | —— | —— |
| Alistipes | 789.44±470.05 | 944.80±560.62 | 1016.11±663.00 | —— | —— | —— |
| **Lachnospiraceae_uncultured** | 673.33±543.83 | 1167.70±1181.11 | 245.00±170.85 | —— | —— | **※** |
| Alloprevotella | 572.44±287.27 | 648.70±496.98 | 691.33±381.86 | —— | —— | —— |
| Bacteroides | 701.67±599.25 | 551.20±262.67 | 383.33±293.33 | —— | —— | —— |
| Faecalibaculum | 564.67±361.31 | 506.90±504.91 | 504.33±769.09 | —— | —— | —— |
| **Desulfovibrio** | 925.44±679.55 | 247.30±195.73 | 7.11±4.65 | —— | **※※※** | **※** |
| Muribaculum | 288.89±181.55 | 522.70±335.81 | 259.22±90.75 | —— | —— | —— |
| Romboutsia | 17.44±14.09 | 626.10±835.83 | 23.44±36.68 | —— | —— | —— |
| [Eubacterium] siraeum group | 71.78±109.43 | 523.60±1048.70 | 52.67±79.68 | —— | —— | —— |
| Lachnoclostridium | 173.89±133.83 | 309.50±320.30 | 96.89±97.79 | —— | —— | —— |
| ^※^*P* < 0.05, ^※※^*P* < 0.01, ^※※※^*P* < 0.01. The P values were calculated using the Kruskal-Wallis test with Bonferroni correction. | | | | | | |
|  |  |  |  |  |  |  |

| **Table S16. Top 20 most abundant species in the Control, DSS and XZ groups.** | | | | | | |
| --- | --- | --- | --- | --- | --- | --- |
|  |  |  |  |  |  |  |
| **Species** | **Mean** | | | **Significance** | | |
|  | **Con** | **DSS** | **XZ** | **Con-DSS** | **Con-XZ** | **DSS-XZ** |
| **Muribaculaceae_uncultured bacterium** | 17662.33±9876.80 | 9436.80±4057.23 | 19523.56±3022.96 | —— | —— | **※** |
| **Akkermansia_uncultured bacterium** | 1093.33±1156.02 | 14189.00±7709.67 | 10769.11±4392.77 | **※※※** | **※※** | —— |
| **Lactobacillus murinus** | 8025.78±7847.25 | 2701.80±1654.72 | 535.44±197.30 | —— | **※※※** | **※※** |
| Ileibacterium valens | 807.67±1579.03 | 4071.50±7287.65 | 3920.00±5267.72 | —— | —— | —— |
| **Dubosiella_uncultured bacterium** | 3503.22±3631.48 | 253.90±271.57 | 2834.44±2729.04 | **※※** | —— | **※※** |
| **Prevotellaceae UCG-001_uncultured bacterium** | 523.78±421.82 | 2664.10±2218.73 | 782.11±453.13 | **※※** | —— | —— |
| Lachnospiraceae NK4A136 group_uncultured bacterium | 770.44±630.94 | 1576.00±1802.42 | 588.33±945.77 | —— | —— | —— |
| **Clostridia UCG-014_uncultured bacterium** | 1637.78±1602.95 | 137.20±66.08 | 1077.44±1154.67 | **※※** | —— | **※** |
| Lactobacillus_Unclassified | 1373.11±1430.22 | 826.80±1087.52 | 437.78±290.43 | —— | —— | —— |
| Limosilactobacillus_uncultured bacterium | 1415.11±1475.59 | 804.80±968.55 | 413.00±237.30 | —— | —— | —— |
| **Lachnospiraceae_uncultured bacterium** | 671.78±543.30 | 1166.70±1180.87 | 244.44±170.17 | —— | —— | **※** |
| Alloprevotella_uncultured bacterium | 572.44±287.27 | 648.70±496.98 | 691.33±381.86 | —— | —— | —— |
| Bacteroides_uncultured bacterium | 629.67±495.14 | 465.90±222.89 | 319.67±255.89 | —— | —— | —— |
| **Alistipes_Unclassified** | 270.56±234.79 | 505.60±286.85 | 547.44±280.77 | **※** | **※** | —— |
| Alistipes_uncultured bacterium | 473.78±281.34 | 371.30±352.69 | 401.00±377.39 | —— | —— | —— |
| **Desulfovibrio_uncultured bacterium** | 925.22±679.36 | 247.20±195.82 | 7.11±4.65 | —— | **※※※** | **※** |
| Lactobacillus reuteri | 582.78±586.60 | 293.70±362.78 | 199.89±123.91 | —— | —— | —— |
| Muribaculum intestinale | 248.56±154.80 | 463.90±300.03 | 222.67±73.94 | —— | —— | —— |
| Faecalibaculum_uncultured bacterium | 323.67±204.24 | 270.40±273.03 | 274.22±433.57 | —— | —— | —— |
| **Ligilactobacillus_uncultured bacterium** | 531.11±465.68 | 200.20±103.32 | 59.33±24.71 | —— | **※※※** | **※** |
| ^※^*P* < 0.05, ^※※^*P* < 0.01, ^※※※^*P* < 0.01. The P values were caculated using the Kruskal-Wallis test with Bonferroni correction. | | | | | | |
|  |  |  |  |  |  |  |

**Supplementary figures**

**
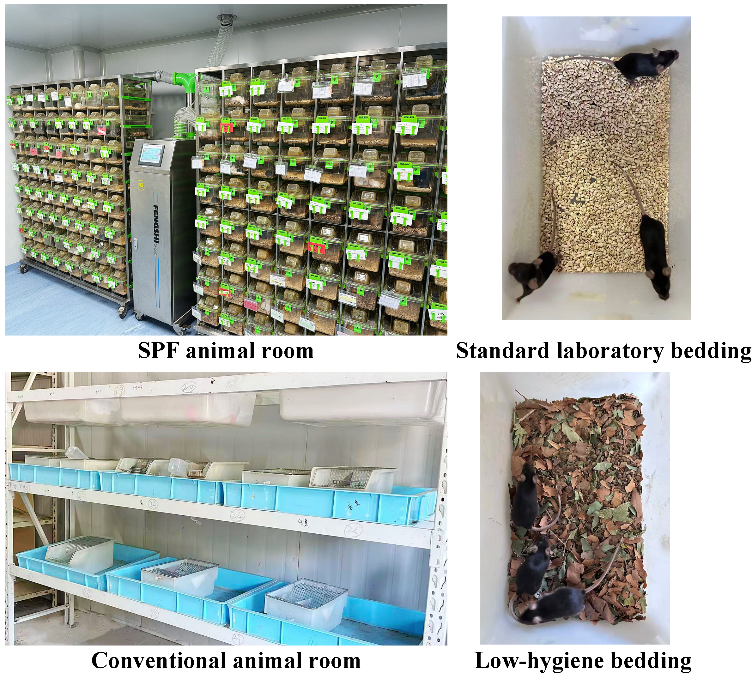
**

**Figure S1.** Representative images of the animal housing environments and bedding materials.

**
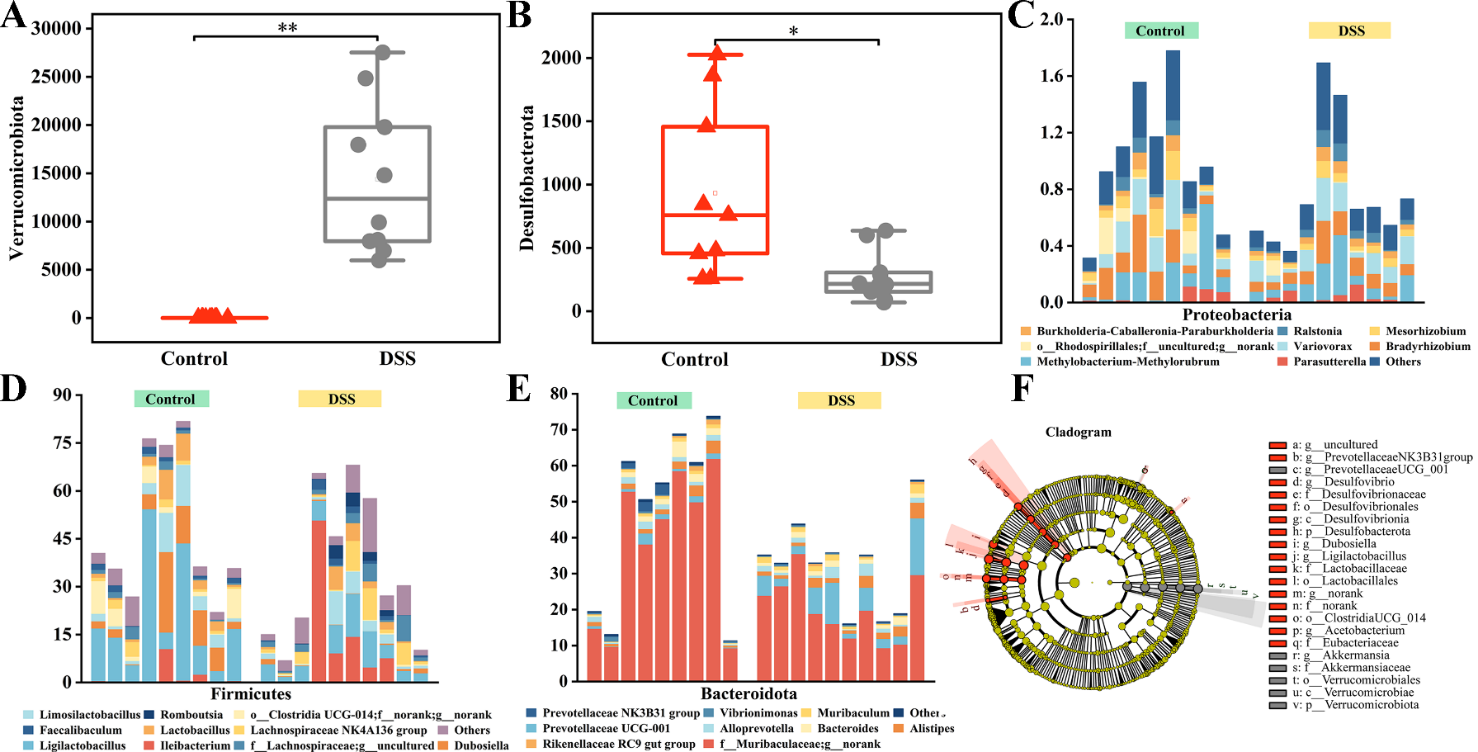
**

**Figure S2. Impact of DSS-induced UC on the gut microbiota in mice.** A-B. Verrucomicrobiota and Desulfobacterota abundance, respectively. C-E. Genus-level compositional changes within the phyla Proteobacteria, Firmicutes, and Bacteroidota, respectively. F. Cladogram of intestinal microbiota.

**
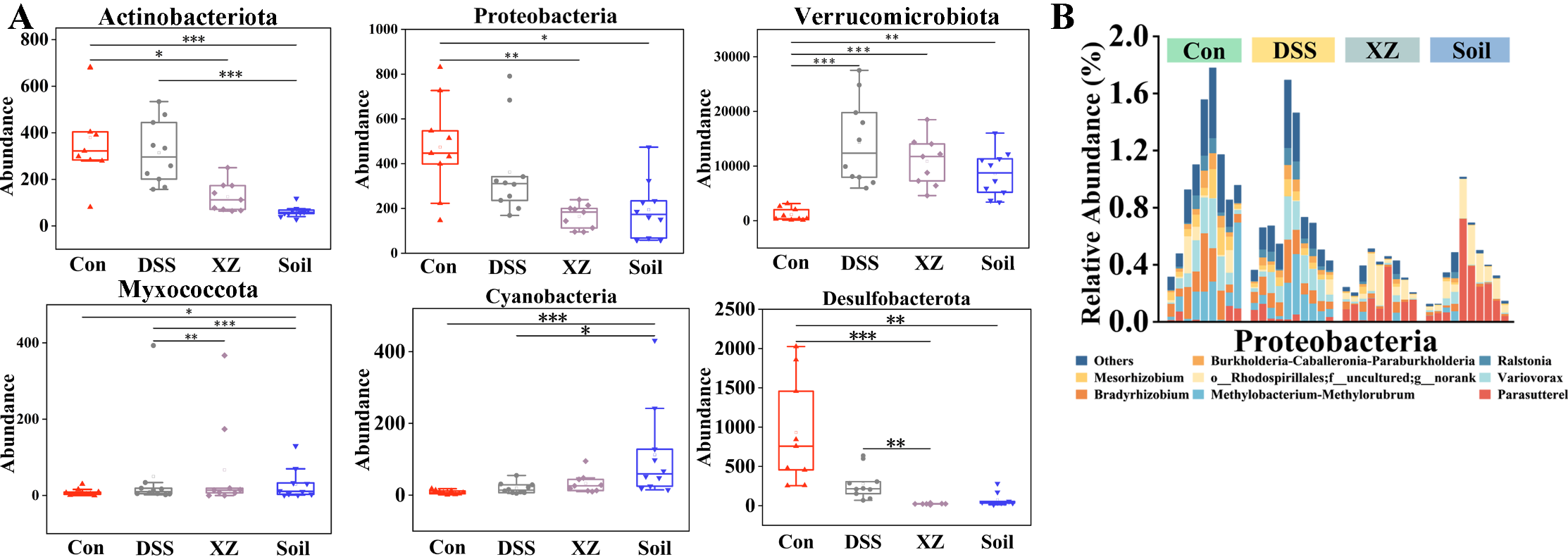
**

**Figure S3.** **Effects of LHE exposure and sterile soil intake on the gut microbiota in UC mice.** A. Abundance of Actinobacteriota, Proteobacteria, Verrucomicrobiota, Myxococcota, Cyanobacteria, and Desulfobacterota. B. Genus-level compositional changes within the Proteobacteria phylum.

**
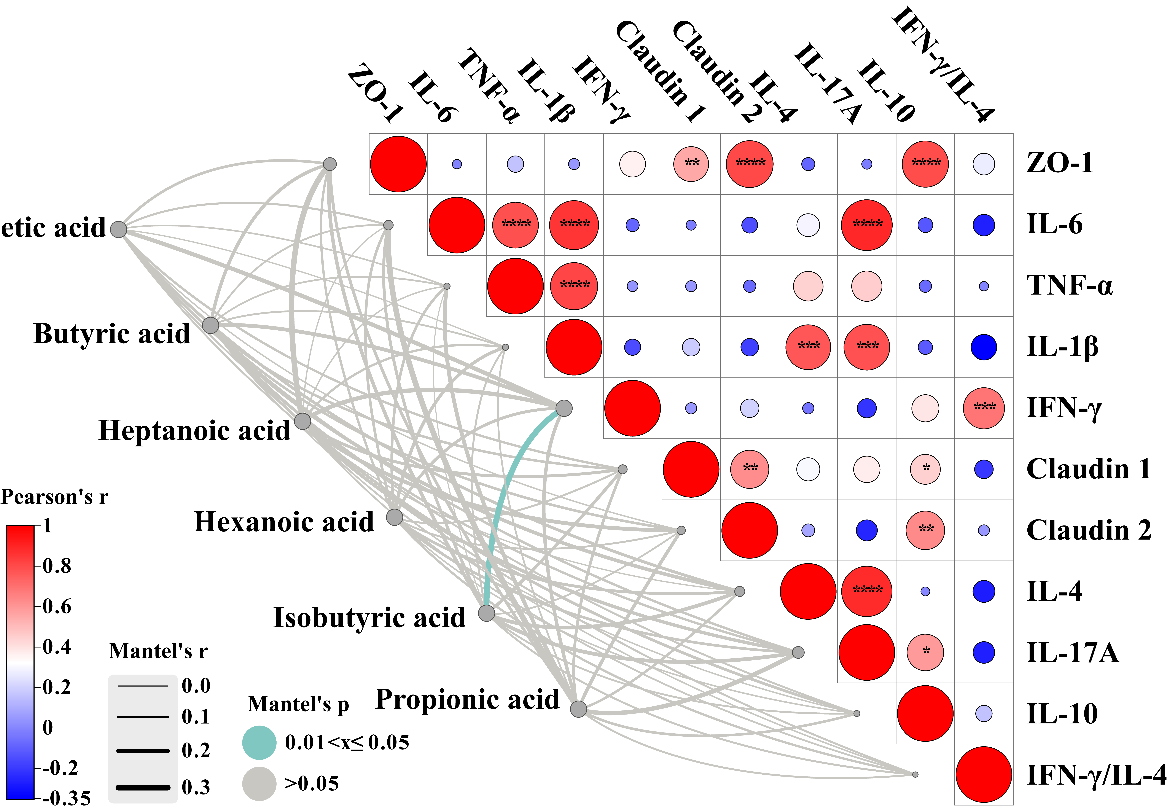
**

**Figure S4. Correlation analyses among SCFAs and immune parameters.** The heatmap shows the results of Pearson correlation analysis of colonic cytokines. The color scale represents Pearson correlation coefficient (* P < 0.05, ** P < 0.01, *** P < 0.001). The chord diagram shows the results of the Mantel test for the correlation between the SCFAs Bray–Curtis distance matrix and immune parameters. Green lines represent significant correlations (cutoff confidence level of 0.05), and the line thickness represents the value of Mantel correlation coefficient.
