# Supplementary figures and images for: Comparable efficacy and mechanisms of sterile soil ingestion versus low hygiene exposure in DSS-induced colitis
Source: Appl Environ Microbiol. 2026 Feb 9;92(3):e02415-25. doi: 10.1128/aem.02415-25 (PMC12997849; doi:10.1128/aem.02415-25)

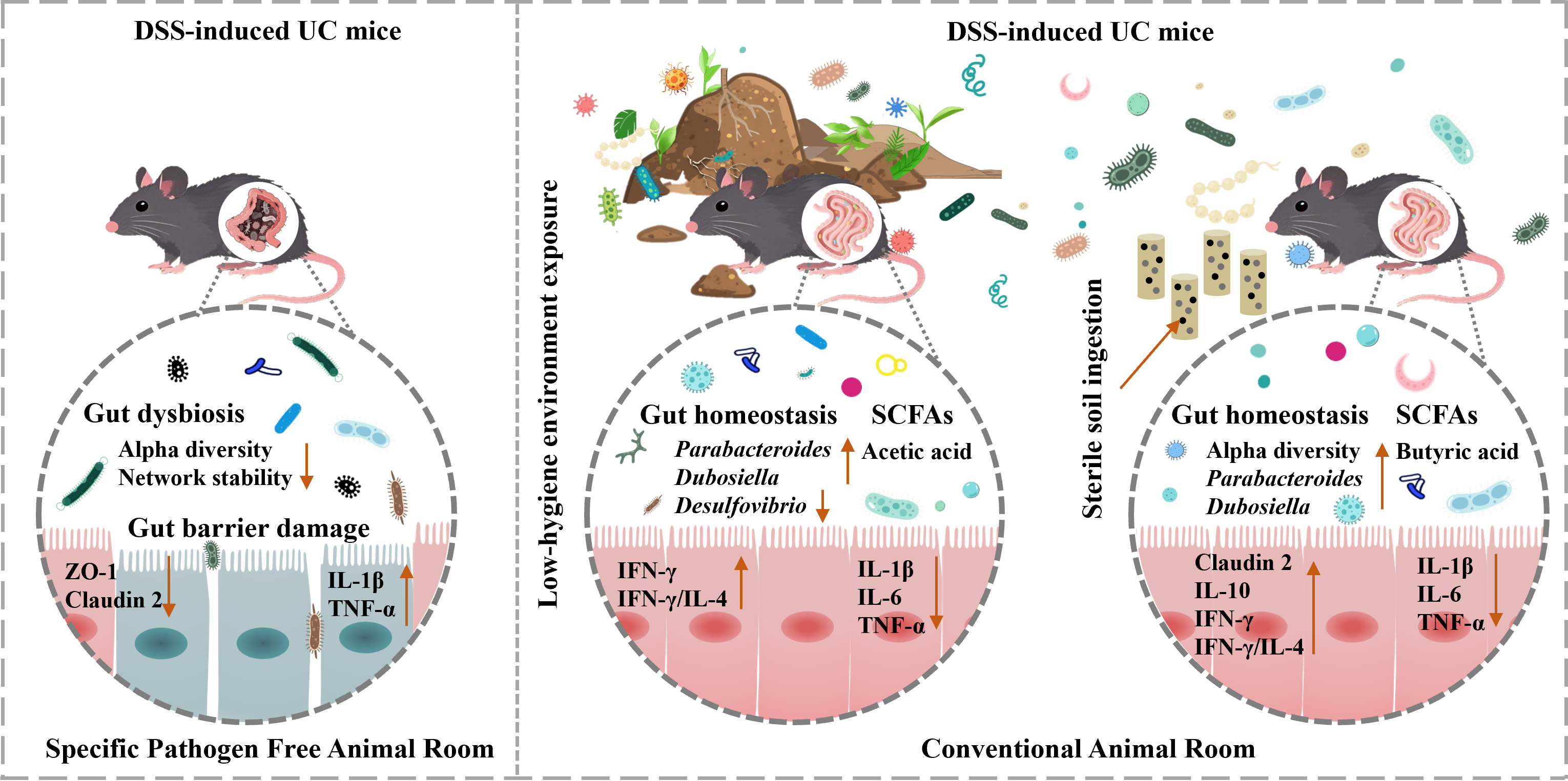

Supplement: Graphical abstract — Visual depiction of the study. [file aem.02415-25-s0002.tif]
